# Export of a *Toxoplasma gondii* Rhoptry Neck Protein Complex at the Host Cell Membrane to Form the Moving Junction during Invasion

**DOI:** 10.1371/journal.ppat.1000309

**Published:** 2009-02-27

**Authors:** Sébastien Besteiro, Adeline Michelin, Joël Poncet, Jean-François Dubremetz, Maryse Lebrun

**Affiliations:** 1 UMR 5235 CNRS, Université de Montpellier 2, Montpelier, France; 2 UMR CNRS 5203, INSERM U661, Université de Montpellier 1 and 2, Montpelier, France; University of Michigan, United States of America

## Abstract

One of the most conserved features of the invasion process in Apicomplexa parasites is the formation of a moving junction (MJ) between the apex of the parasite and the host cell membrane that moves along the parasite and serves as support to propel it inside the host cell. The MJ was, up to a recent period, completely unknown at the molecular level. Recently, proteins originated from two distinct post-Golgi specialised secretory organelles, the micronemes (for AMA1) and the neck of the rhoptries (for RON2/RON4/RON5 proteins), have been shown to form a complex. AMA1 and RON4 in particular, have been localised to the MJ during invasion. Using biochemical approaches, we have identified RON8 as an additional member of the complex. We also demonstrated that all RON proteins are present at the MJ during invasion. Using metabolic labelling and immunoprecipitation, we showed that RON2 and AMA1 were able to interact in the absence of the other members. We also discovered that all MJ proteins are subjected to proteolytic maturation during trafficking to their respective organelles and that they could associate as non-mature forms *in vitro*. Finally, whereas AMA1 has previously been shown to be inserted into the parasite membrane upon secretion, we demonstrated, using differential permeabilization and loading of RON-specific antibodies into the host cell, that the RON complex is targeted to the host cell membrane, where RON4/5/8 remain associated with the cytoplasmic face. Globally, these results point toward a model of MJ organization where the parasite would be secreting and inserting interacting components on either side of the MJ, both at the host and at its own plasma membranes.

## Author Summary

A unique feature of apicomplexan parasites is the formation of an intimate contact between the apex of the parasite and the host cell membrane called the moving junction that moves along the parasite during invasion. Proteins originated from two distinct secretory organelles, the microneme for AMA1 and the rhoptry neck for RON2/4/5 proteins, are associated to form the junction. Here, we have furthered the characterization of the MJ complex by describing RON8, an additional protein component. AMA1 has previously been shown to be inserted into the parasite membrane upon secretion. Our study demonstrates that all the RON proteins are translocated into the host cell, where RON4/5/8 remain associated with the cytoplasmic face of the host cell plasma membrane. Furthermore, we identified a privileged interaction between transmembrane MJ proteins AMA1 and RON2 *in vitro*. Overall, this led us to propose the first model describing the putative MJ organisation at the interface between the host cell and *Toxoplasma*. In this original concept, the parasite would export its own receptor (RON2) and ligand (AMA1) on either side of the MJ.

## Introduction

Invasion by Apicomplexa is an essential step of the pathologies associated with these protozoan parasites that include *Plasmodium* spp., the causative agents of malaria, as well as *Toxoplasma gondii*, responsible for human and animal toxoplasmosis. The invasive stages of these parasites share a highly conserved architecture, including a cytoskeleton-associated original pellicular complex, and two types of vesicular apical organelles (micronemes and rhoptries) that participate to the invasion process through the exocytosis of their contents in a sequential manner [1]. Host cell invasion has been well described at the ultrastructural level, but the precise molecular interactions and the specific role of the exocytosed parasite proteins are still poorly understood. Proteins located on the surface of the parasite probably mediate the initial interaction with the target cell. This is followed by an intimate contact between the apical tip of the parasite and the host cell membrane, called the moving junction (MJ) [2]. This singular structure, likely linked to the subpellicular cytoskeleton motor of the parasite, might serve as a support to propel the parasite into the parasitophorous vacuole (PV) that forms inside the host cell. To do so, the MJ rapidly turns into a ring that is moved backward along the parasite during invasion and ends up at the posterior end of the invaded parasite at the end of the process. Despite a number of investigations having led to the discovery of a variety of putative parasite adhesive molecules secreted from micronemes, and of an original acto-myosin based motor for gliding motility [3], the process of invasion itself (i.e. MJ-dependent host cell entry), remains a major conundrum. Indeed, although the morphological features of the process have been described 30 years ago [2], the MJ was, up to a recent period, completely unknown at the molecular level. The major reason for this was its transient nature, since host cell invasion is a very rapid process (a few seconds), and therefore isolating the structure was not possible.

Rhoptries are elongated organelles composed of a bulbous body that tapers into a thin duct-like neck. Rhoptries empty their contents apically during the invasion process, after microneme exocytosis, and their contribution to invasion was considered mostly as providing building material for the developing PV, since proteins of the bulb of the rhoptry (ROPs) were found associated with the nascent vacuole membrane (for a review see [4]). Recently, an unexpected function of the rhoptries in MJ formation arose from the discovery that one rhoptry neck protein (RON4) was associated to the MJ [5],[6]. It was proposed that MJ formation would derive from a cooperation between i) newly discovered RONs located in the rhoptry neck and ii) the micronemal protein AMA1 [5]. Numerous lines of evidence suggest that the conserved AMA1 protein plays a central role during invasion of Apicomplexa. For instance, AMA1 has been shown to be essential for *Plasmodium* merozoites and *Toxoplasma* tachyzoites [7],[8]. In *T. gondii*, AMA1 and RON4 have been found to be associated in a complex *in vitro* and they localize precisely at the MJ during cell invasion, although a direct association of the two proteins has not been demonstrated *in vivo* [5],[6].

The isolation of RON4 from parasite extracts by affinity purification led us to the simultaneous purification of the rhoptry neck protein RON2 and protein TwinScan_4705 (annotated also 583.m00636) [6], which was later shown to be also a RON (RON5, P. Bradley personal communication). Like AMA1, RONs are conserved throughout the Apicomplexa including *Plasmodium spp.*, and they are not found outside this phylum. AMA1 and RONs are stored in two distinct compartments that release their content sequentially during invasion. Cross-linking experiments on invading parasites showed that the interaction of AMA1 with RONs takes place during invasion and is not the result of non-specific or indirect binding occurring in the parasite lysate during IP [5]. One intriguing question is how the micronemal protein AMA1 and the complex of rhoptry neck proteins RON2/RON4/RON5 avoid interacting in the secretory pathway. Another important question is how these proteins are organized at the MJ. The microneme protein AMA1 has been characterized structurally and appears to be translocated as a type-1 transmembrane (TM) protein in the tachyzoite plasma membrane [9],[10]. On the contrary, the topology of the RONs at the MJ is still obscure and several important questions remain unanswered. Are RONs directly or indirectly linked to the parasite surface? Could they be binding to a host cell receptor or, as we speculated previously [6], are they directly inserted into the host cell membrane to serve as a receptor for AMA1?

Here, we describe an additional partner of the previously characterized AMA1/RON2/4/5 complex named RON8. We also show that the complex may be assembled as pro-proteins but that a distinct timing of biosynthesis between MICs and RONs precludes the association of RONs with AMA1 before secretion. Furthermore, we demonstrate that RONs are exported to the host cell membrane, RON/4/5/8 being exposed to the host cell cytosol and RON2 being probably an integral membrane protein that displays a privileged interaction with AMA1. These results provide an important clue to understand how such a crucial structure for the invasive and developmental processes of the parasite is built and organized.

## Results

### Identification of RON8 as an additional partner of the AMA1-RON2/4/5 complex

**Figure 1:**
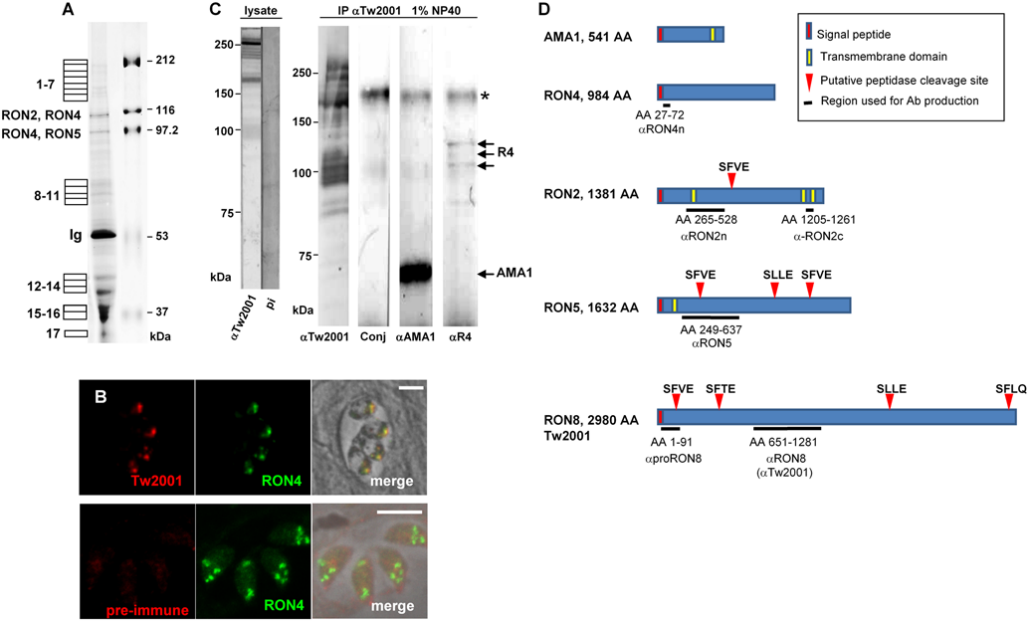
Identification of an additional moving junction complex member, RON8. (A) Isolation of RON4-associated proteins by immuno-affinity column. Eluate from the purification of a tachyzoite lysate on T5 4H1 immunosorbent was resolved by 8% SDS-PAGE and stained with colloidal blue. Bands taken for mass spectrometry analysis are numbered on the left, and peptides identified are listed in Table S1. Bands previously identified as RON2, RON4, and RON5 are indicated, as well as band corresponding to immunoglobulin (Ig). Molecular mass standards are indicated on the right. (B) Co-localisation by IFA of RON8 (Tw2001) with RON4 to the rhoptry neck within intracellular tachyzoites fixed and permeabilized with methanol. The absence of reactivity of the pre-immune serum is presented in the lower panel as a negative control. (C) Lysate: Western blot with anti- Tw2001 of a *Toxoplasma* lysate run in non-reduced condition. (pi: pre-immune serum). IP αTw2001: Co-IP of RON8, RON4, and AMA1 using anti-RON8 antibody. Proteins isolated on an anti-RON8 immuno-affinity column were separated on SDS-PAGE in reduced condition, transferred to nitrocellulose and probed with anti-mouse secondary antibody conjugate alone (Conj), with T5 4H1 mAb (anti-R4) or with CL22 mAb (anti-AMA1). Proteins of interest are shown by arrows; * denotes a non-specific band. Molecular mass standards are indicated on the left. (D) Schematic representation of the MJ proteins with predicted features such as TM domains, as predicted by ConPred II (http://bioinfo.si.hirosaki-u.ac.jp/~ConPred2/), and putative SUB2 cleavage sites (red arrowheads). Regions used as recombinant proteins for the production of the specific antibodies described in Text S1 are underlined.

In order to further refine the molecular characterization the MJ complex of *T. gondii*, we searched for additional proteins co-immuno-purified (IP) by the anti-RON4 antibody matrix, as previously described [6]. The RON4-associated proteins were subjected to mass spectrometric analysis. As in our first analysis, we detected two principal bands at ∼120 kDa and ∼100 kDa, which corresponded to RON2, RON4 and RON5 (Figure 1A). In addition to the proteins of the MJ already known to be associated with each other (RON2, RON4, RON5 and AMA1), mass spectrometry analysis identified peptides from proteins that are described in Table S1. Peptides from proteins originated from the secretory organelles involved in invasion (microneme and rhoptry) have retained our attention. First, peptides from two microneme proteins MIC1 [11] and MIC3 [12] were detected. However, Western blot and reverse IP analysis using anti-MIC3 or anti-MIC1 antibodies did not confirm a specific interaction of these proteins with the MJ complex proteins (data not shown). Second, we found peptides from TwinScan_0092 (80.m02161) and TwinScan_2001 (541.m00141), two predicted *Toxoplasma* proteins that had also been detected in the proteomic analysis of the rhoptries [13]. TwinScan_0092 predicts a protein of 49 kDa that is not localising at the MJ but was instead found to be a new dense granule protein [14]. Concerning TwinScan_2001, a previous study using an antibody raised against a specific peptide had localised it to the apicoplast by IFA [13], although it does not possess any *bona fide* plastid-targeting element in its amino acid sequence. We then decided to reassess its subcellular localization by generating a specific polyclonal antiserum directed against a recombinant TwinScan_2001 protein corresponding to the central part of the protein (Figure 1D). This antibody (anti-Tw2001) reacted on Western blot with a major band of about to 250 kDa and several minor bands of lower molecular mass (Figure 1C), that were also detected with an additional serum raised against another region of the protein (not described here), but were absent when probed with the pre-immune serum (Figure 1C, Figure S1A). By IFA, the anti-Tw2001 serum recognized an antigen co-localized with RON4 in intracellular parasites (Figure 1B), suggesting that TwinScan_2001 was a new rhoptry neck protein that we named RON8.

To further verify that RON8 is associated with the AMA1/RON2/4/5 complex, we performed an IP using the anti-Tw2001 serum (referred to as anti-RON8 throughout the manuscript), as described previously for RON4 [6] and showed co-purification of RON8, AMA1 and RON4 (Figure 1C). The formation of a stable complex in 1% NP40 and 1 M NaCl conditions, containing RON2/4/5/8 and AMA1, was further confirmed by co-IP of all members after affinity chromatography using either of the specific anti-RONs (data not shown).

The complete coding sequence of *RON8* was determined (GenBank accession number ACK57540) and showed that it coded potentially for a 2979 amino acids-long protein, with a theoretical molecular mass of 329 kDa. A putative signal peptide was found at position 1–29. PROSITE search yielded no obvious sequence motifs. A search of the GenBank non-redundant database and ApiDB showed that RON8 is unique to *Toxoplasma* and *Neospora* among Apicomplexa (in contrast to other MJ proteins) and is not found in other organisms.

### RON2, RON4, RON5, and RON8 are all present at the MJ during invasion

We have previously shown that RON4 is associated with the MJ during invasion [6], here we examined if RON2, RON5 and RON8 would also follow the MJ. We first generated antisera specific of RON2, RON4 and RON5. For RON2, two sera were prepared against different regions of the protein produced as recombinant proteins named RON2n and RON2c (see Figure 1D). An anti-serum against the N terminal part of RON4 (RON4n) was also produced.

**Figure 2:**
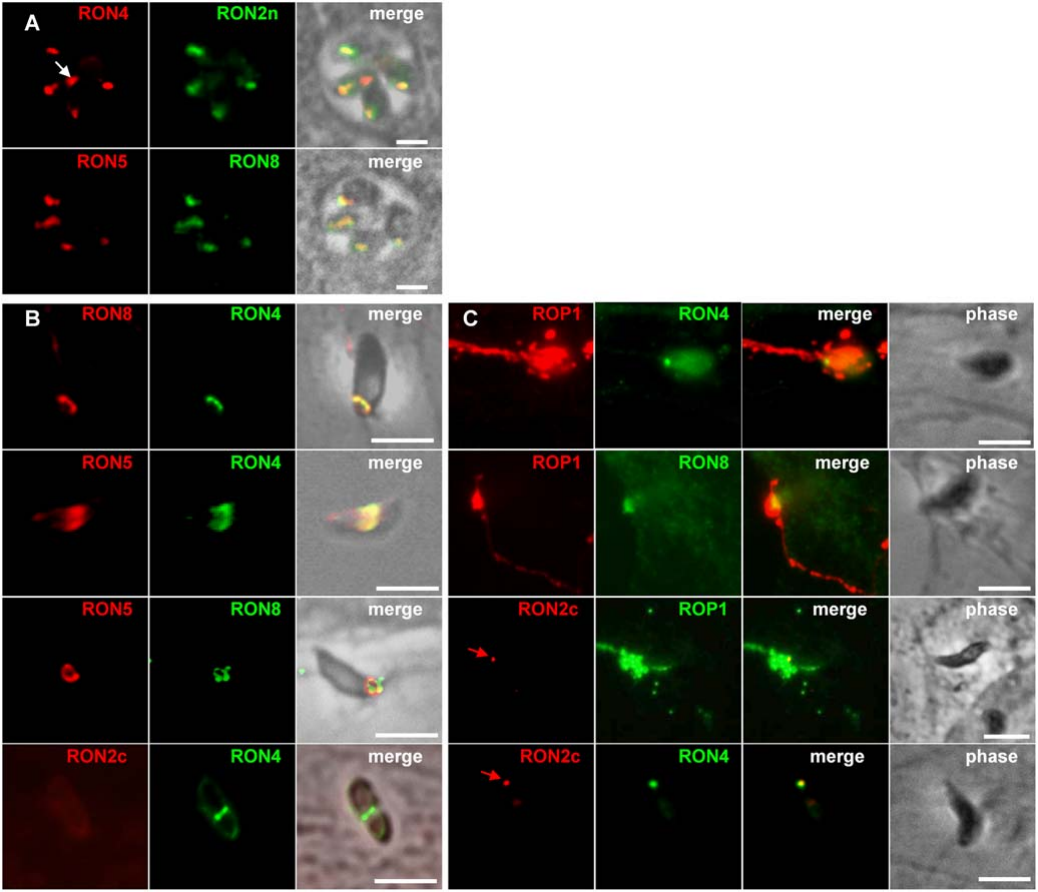
IFA detection of RON2, RON4, RON5, and RON8 in the rhoptry neck, and at the MJ. Scale bar = 5 µm. (A) Co-localisation of RON2,4,5,8 to the rhoptry neck within intracellular tachyzoites growth overnight on HFF cells, fixed, and permeabilized with methanol. Identical results were obtained with anti-RON4n and anti-RON2n (not shown). Arrow indicates RON4 present in the PV as shown previously [6]. (B) RON8, RON5, and RON4, but not RON2, can be detected at the ring of the MJ during invasion. Phase contrast and IFA of parasites show that mAb anti-RON4, polyclonal anti-RON5, and polyclonal anti-RON8 label the ring corresponding to the MJ in invading parasites permeabilized with 0.05% saponin. The ring was not detected using anti-RON2c (lower panel). (C) As other junctional RONs, RON2 can be detected at the junction in cytD-treated tachyzoites attached to the surface of the host cell. Evacuoles expanding within the host cell cytosol from the site of parasite attachment are labelled with anti-ROP1. RON2 (labelled with anti-RON2c, arrow), RON4, and RON8 are found associated with the tip of the parasite; RON2 is shown to be colocalizing with RON4 in the lower panel.

The specificity of the sera was first analyzed by Western blot on whole tachyzoite lysates (Figure S1A). All sera recognized in non-reduced condition a band at about the predicted size (RON2: 155 kDa,, RON5: 179 kDa, RON8: 329 kDa). No detection was observed with pre-immune sera. In reduced condition, the anti-RON2c, anti-RON2n, anti-RON4n, and anti-RON5 recognized proteins that migrated faster, indicating that, as previously shown for RON4 ([6] and Figure S1A), RON2 and RON5 are also sensitive to reduction of disulfide bonds (discussed later). The sera were also analyzed by IFA on intracellular parasites. As shown in Figure 2A, all the anti-RONs labelled the neck of the tachyzoites rhoptries, as indicated by co-localisation with RON4 and RON8. Throughout the study, the anti-RON4 T5 4H1 [15] and the anti-AMA1 CL22 mAbs were used systematically, except when specified.

On invading parasites, in permeabilization conditions optimized to detect only the material secreted by the parasite [1], we showed that anti-RON5 and anti-RON8 recognized exclusively the characteristic ring-shaped MJ (Figure 2B). In contrast, in these conditions both anti-RON2 antibodies failed to react (Figure 2B, lower panel). We have shown previously that when the PVM has pinched off the host cell, the MJ can still be detected at the posterior pole of the parasite for a few hours and is characterized by a dot-like signal with anti-RON4 mAb [6]. Again, we showed using specific antibodies that RON5 and RON8 could be found together at the same location, but not RON2 (data not shown). Since cytochalasin D (Cyt-D, an inhibitor of actin polymerization)-treated parasites form a “static” junction that is labelled by anti-RON4 [6] but not translocated to the posterior end of the tachyzoites [16],[17], we tested if all the RONs could be immunolocalized at the junction in these conditions. We found that after Cyt-D treatment, in addition to RON4 [6], all the proteins of the complex, this time including RON2 (yet only with the anti-RON2c antibody), could be detected at the same location (Figure 2C). The detection of RON2 in these conditions could be explained by the fact that the Cyt-D treatment had improved the accessibility of the protein to the antibody, either because it destabilised some link of RON2 with Cyt-D sensitive structures of the host or, more simply, that it blocked the junction in an early stage where the protein is more accessible. Overall, this is strengthening the idea that RON2, RON4, RON5 and RON8 are present together in the MJ complex during invasion.

### RON2 and AMA1 can interact together in the absence of RON4/5/8

**Figure 3:**
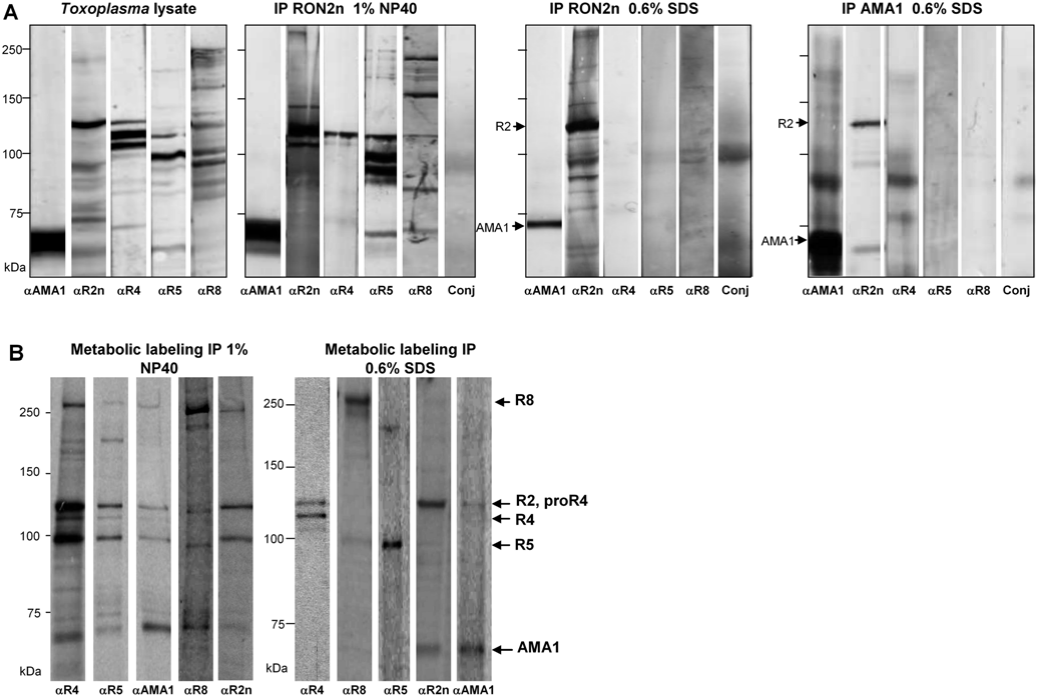
Interactions between members of the junctional complex. (A) Western blot analysis in reduced condition of MJ members co-immunoprecipitated in various detergent conditions (1% NP40 or 0.6% SDS) with the antibody mentioned on top and probed with the antibodies mentioned below or probed with secondary antibody conjugate alone (Conj). The first panel corresponds to the *Toxoplasma* lysate used for the IP and probed with the antibody mentioned below. Molecular mass standards for all the panels are indicated on the left. (B) Co-immunoprecipitation in two detergent conditions of MJ proteins after metabolic labelling, with specific antibodies mentioned below. Metabolic labelling was done with [^35^S] methionine/cysteine for 15 min on intracellular parasites followed by one hour of culture without radioactivity. Cells were lysed either in 1% NP40 (left panel) or 0.6% SDS (right panel), and proteins were immunopurified using specific antibodies directed against each member of the MJ complex. The IP products were revealed by autoradiography after separation by SDS-PAGE in reduced conditions. MJ proteins positions are shown on the right. Although the profiles in NP40 conditions appear similar, these lanes migrated on independent gels, thus the positions of the RONs are not exactly the same and the molecular mass standards are only indicative.

The generation of antisera against the individual members of the MJ complex allowed us to analyse more precisely the RONs and their interactions by IP using different conditions for solubilization of the parasite. After lysing the parasites in 1% NP40 (the condition used to immunopurify the complex [6]), all members of the complex were recovered using each of the anti-sera available, as exemplified in Figure 3A with an IP using the anti-RON2n serum followed by Western blot analysis of each member of the MJ complex. We then tested the stability of the complex upon tachyzoite lysis in 0.6% SDS followed by heat denaturation. In these conditions, only the interaction between AMA1 and RON2 was maintained after IP with either anti-RON2n or anti-AMA1 (Figure 3A) and no interaction between the others RONs was observed (ie using anti-RON4n, anti-RON5 and anti-RON8, Figure S1B). These results were confirmed by comparing the profiles obtained after metabolic labelling of intracellular parasites with [^35^S]-methionine/[^35^S]-cysteine, and lysis in either 0.6% SDS or 1% NP40, followed by IP with anti-RONs antibodies (Figure 3B). A similar profile in which all members of the complex were detected was obtained after IP in 1% NP40 whatever the antibody used (left panel). In contrast in 0.6% SDS, while the anti-RON4, anti-RON5 and anti-RON8 immunopurified only the corresponding protein, the anti-RON2n and anti-AMA1 immunopurified both AMA1 and RON2 (right panel). These results clearly indicated that the whole complex was not maintained in 0.6% SDS, but that AMA1 and RON2 proteins interact together particularly strongly, independently of the other MJ proteins.

### RONs are subjected to proteolytic maturation and processed in the pre-rhoptries

**Figure 4:**
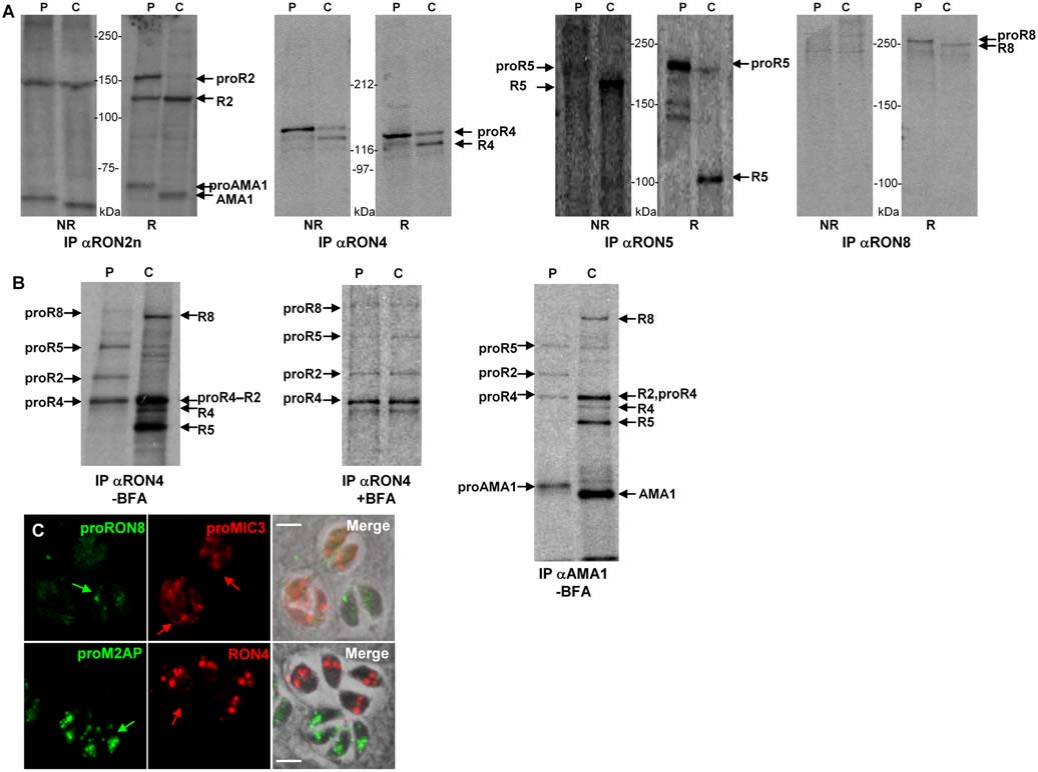
MJ proteins are processed and can associate as non-mature forms. (A) Pulse-chase metabolic labelling and IP with specific antibodies showing processing of MJ proteins. *T. gondii*-infected fibroblasts were labelled for 15 min with [^35^S] methionine/cysteine and either harvested (P, pulse) or chased for 1 h (C, chase). Then, the 0.6% SDS lysates were immunoprecipitated with the antibody indicated below, and products were run in SDS-PAGE in reduced (R) or non-reduced (NR) conditions before autoradiography. Proteins and their pro-forms are annotated. Molecular mass standards are indicated. (B) Similar analysis in the presence of BFA shows that maturation occurs in a post-Golgi compartment and that non-mature forms are recovered together by IP. Pulse-chase was done as in (A), with or without BFA, and IP were performed on cells lysed in 1% NP40. In the presence of BFA, the same profile was obtained in the pulse and the chase, indicating that pro-RONs can assemble as immature forms. Moreover, proAMA1 could also associate with the RONs complex (lane corresponding to the pulse in the right panel). (C) Biosynthesis of RONs and MICs are asynchronous. Double IFA with anti-proRON8 and anti-proMIC3 antibodies (upper panels) or anti-RON4 and anti-proM2AP antibodies (lower panels) on intracellular parasites fixed with 4% PAF and permeabilized with Triton X-100 label tachyzoites in distinct vacuoles (coloured arrows), at different stages of endodyogeny.

Most *T. gondii* rhoptry bulb proteins described so far are synthesized as pro-proteins that are subjected to removal of their N-terminal pro-region by proteolytic cleavage during traffic to the organelle. To determine whether the RONs are also processed, we studied their biosynthesis and maturation by pulse-chase metabolic labeling with [^35^S]-methionine/[^35^S]-cysteine followed by IP with anti-RONs antibodies (Figure 4A). The infected cells were lysed and boiled in 0.6% SDS to avoid co-purification of the whole complex.

For RON2, after 20 minutes of pulse, a protein of ∼150 kDa (reduced) was immunoprecipitated, which is consistent with the predicted size of RON2 after the removal of the signal peptide; a minor band was also found at ∼120 kDa. After one hour of chase, the 150 kDa disappeared and the 120 kDa band was the major one detected. A 65 kDa band after chase and a slightly slower migrating one in the pulse corresponded to AMA1 (as described above) and proAMA1 respectively (see below). In non-reduced condition, the 150 kDa band was detected both in pulse and chase fractions, indicating that RON2 is processed and that the two fragments might be linked by internal disulfide bonds (several cysteines are present in both fragments). For RON5, after 20 minutes of pulse, a major protein of ∼180 kDa (unreduced) was immunoprecipitated, which is consistent with the predicted size of the protein. After one hour of chase, the 180 kDa product almost disappeared and a ∼150 kDa band was detected instead. In reduced condition, the 180 kDa form was also detected in pulse, while a ∼110 kDa form was immunoprecipitated after one hour of chase. A band of ∼30 kDa was also detected by Western blot on whole tachyzoites in reduced condition (data not shown) and was recovered by IP (Figure S2). These results indicated that RON5 is cleaved at least at two sites, one processing event resulting in removal of a pro-sequence (as for many ROP proteins), and another processing event yielding two polypeptides possibly bound by a disulfide bond (as for RON2). Concerning RON8, a processing event was also detected in reduced and non-reduced conditions, indicating that RON8 was also subjected to removal of a pro-sequence. Pulse-chase experiment for RON4 also showed that it is expressed as a pro-protein (∼120 kDa reduced condition and ∼145 kDa unreduced) that is cleaved to yield a mature protein of ∼110 kDa (reduced) or ∼120 kDa (unreduced). One additional minor band of lower molecular mass was also sometimes present. The persistence of the immature form of RON4 after one hour of chase indicated that RON4 was only partially matured. This could be linked to the fact that, as shown before [6],[13], part of RON4 is secreted in the PV (arrow in Figure 2A) and therefore avoids the rhoptry-specific processing compartment. Serendipitously, the generation of a transgenic parasite cell line expressing a Ty-tagged version of RON4 (see Text S1) that was, for unknown reasons, entirely secreted in the vacuolar space (Figure S3A) and remained entirely unprocessed (Figure S3B), strengthened this hypothesis.

In order to determine in which compartment the RONs were processed, we then generated antibodies directed against the RON8 pro-peptide. As for all rhoptry proteins described so far, this latter was assumed to be located N-term and cleaved by the protease TgSUB2 [18]. Three putative TgSUB2 cleavage sites were found in RON8, two in RON5 and one in RON2 (Figure 1D). We therefore raised antibodies against a peptide spanning RON8 AA 1-91, located before the first SFVE motif of the RON8 sequence (Figure 1D). IP using anti-proRON8 demonstrated the specificity of the anti-proRON8 for the immature form of RON8 (Figure S4A), whereas IFA showed reactivity restricted to the characteristic pre-rhoptry compartment (Figure S4B), which corresponds to the nascent rhoptries of daughter parasites during endodyogeny. The cleavage of all RONs beyond the ER was showed by pulse/chase analysis in the presence of the Golgi transport-inhibiting drug brefeldin A (BFA), pro-RONs remaining the only forms of the proteins at the end of the chase (data not shown and Figure 4B).

### RONs and AMA1 can interact as pro-proteins

Since RONs undergo a proteolytic maturation, we analyzed if they could bind as immature proteins or if processing was required for this binding. To this end, we analyzed the MJ complex by pulse-chase experiments, followed by IP in lysis conditions known to preserve the association of the complex (using 1% NP40). As shown in Figure 4B, after a 15 min pulse, the immature forms of RON2, RON8 and RON5 could be recovered after IP with the anti-RON4 monoclonal. Similarly, and as a complementary approach, IP of the pro-forms of the other MJ partners was achieved using anti-RON2n, anti-RON5 or anti-RON8 (data not shown). To confirm these data, we checked for the association of the complex in BFA-treated cells that would express only immature radiolabeled RONs. As expected, in presence of the drug, proRON2 (150 kDa), proRON8 (329 kDa), proRON5 (180 kDa) and proRON4 (120 kDa, reduced) were the only species found at the end of the chase and co-precipitated together (Figure 4B). Pulse-chase with anti-AMA1 serum confirmed that AMA1 was also processed during traffic [19], and that proAMA1 could also associate to pro-RONs (Figure 4B, right panel), a result which was also observed in pulse chase experiment using anti-RON2n (Figure 4A, left panel).

Overall, these results show that all known members of the AMA1-RONs complex could associate together as pro-proteins *in vitro*.

### Asynchronous biosynthesis of RONs and MICs precludes their interaction in the biosynthetic pathway

Since AMA1 and RON2/4/5/8 could interact together as pro-proteins *in vitro* and would follow the same secretory pathway (i.e. rough ER and Golgi apparatus) before being packaged in their respective compartments in the parasite, we raised the question of how the micronemal protein AMA1 and the complex of rhoptry neck proteins RON2/4/5/8 could avoid interacting before secretion. One possibility would be that they are synthesized sequentially and never coexist in the same compartment. We checked this hypothesis by IFA. Since maturation of both MICs and RONs occurs with rapid kinetics (MICs mature within 15-60 min, [20] and Figure 4A) and the pro-sequence is only transiently detected by IFA, detection using anti-propeptide antibodies faithfully reflects the timing of their synthesis. We therefore performed double IFA with the rabbit polyclonal anti-pro-RON8 and the antiserum raised by Hehl et al. [9] against a peptide corresponding to the AMA1 pro-sequence (data not shown). Unfortunately, this anti-proAMA1 serum gave a very low signal/noise ratio and no significant data could be obtained with this probe. Since we had previously studied the fate of other microneme prodomains and showed for example that 80% of the parasites co-expressed both pro-forms of microneme proteins M2AP and MIC3 simultaneously [21], we reasoned that AMA1 could follow the same scheme and therefore decided to use the mouse anti-proMIC3 and the rabbit anti-proM2AP sera instead. Interestingly, no colocalization of proRON8 and proMIC3 was ever found and both markers were only observed simultaneously in ∼7% of the parasites (±2%, mean±SEM of 3 independent experiments; usually in very large parasites in mid-stages of endodyogeny) (Figure 4C, upper panels). We then took advantage that mAb anti-RON4 did not label the mature rhoptries but only the pre-rhoptries of dividing parasites upon formaldehyde fixation and triton permeabilization [6], to compare the timing of synthesis of RON4 with that of M2AP using rabbit anti-proM2AP. Dual staining using rabbit anti-proM2AP serum and anti-RON4 showed that pre-rhoptry RON4 staining was almost never associated with a proM2AP staining in the same parasite (8.1%±3%, mean±SEM of 3 independent experiments) and apparently not in the same compartment, while RON2, RON5 and RON8 were systematically detected simultaneously with RON4 in pre-rhoptries in the same conditions (data not shown), confirming that RONs and MICs biosynthesis are asynchronous. This would allow MICs and RONs to reach their correct destination without interacting before secretion.

### The RONs complex is targeted to the host cell membrane during invasion

As reported above, RON 2, 4, 5, and 8 are found at the MJ by IFA, but their precise location respectively to the parasite or host cell membrane is not known. We thus sought to determine which membrane these RONs were associated with.

**Figure 5:**
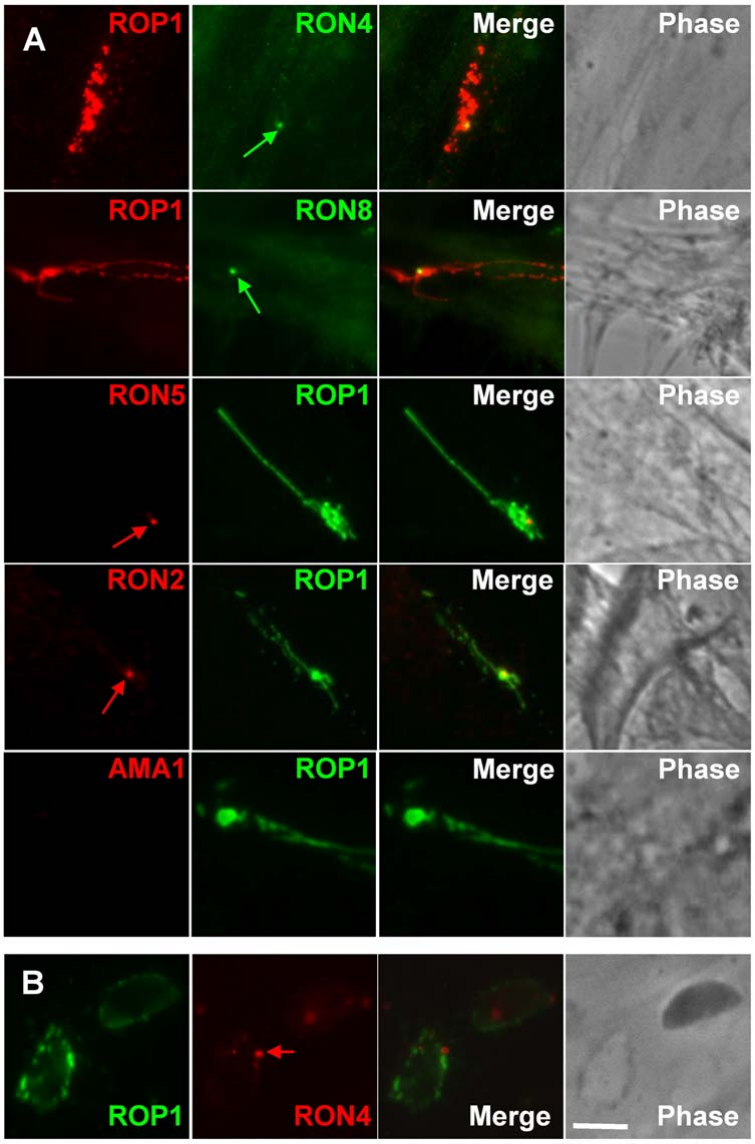
Association of RONs with the host cell plasma membrane. (A) A RON spot persists on cells after abortive interaction with a parasite in the presence of Cyt-D. Dual IFA were done with anti-RONs and anti-ROP1 on invading parasites stopped by Cyt-D, fixed, and permeabilized with saponin. The anti-ROP1 labeled the evacuoles network resulting from the secretion of rhoptries. The absence of a parasite at the site of secretion of ROP1 and the detection of a single dot with all of the anti-RONs (arrowed), but not with anti-AMA1, indicated that RONs could associate with the host cell membrane. (B) IFA of HFF cells pulse-invaded for 15 min, permeabilized with saponin, and incubated with both the rabbit serum anti-ROP1 and the mAb anti-RON4. The image shows two vacuoles labelled with anti-ROP1, one containing a parasite (right) and the other being empty (left). The arrow indicates the presence of the junctional protein RON4 on the empty vacuole. Scale bar = 5 µm.

First, we observed that in IFA on parasites invading cells in the presence of Cyt-D, the rhoptry protein ROP1 was sometimes detected on cells in the absence of any surrounding parasite (Figure 5A), suggesting that it would either correspond to an abortive invasion after secretion of the rhoptry content or that the parasite has been mechanically removed by the washes during the experimental procedure. Abortive invasion has been previously documented in a recent mathematical model showing that approximately 55% of the parasites detach within 5 min of initial attachment, but this paper did not conclude on whether the moving junction was built or not before detachment [22]. We thus assessed the presence of RONs in this particular situation. Dual IFA showed that all RONs could usually be detected as a punctuate signal, near the point of contact where the rhoptry content had apparently been initially discharged, as detected by anti-ROP1 staining in the host cell permeabilized with saponin (Figure 5A). RON2 was only detected with anti-RON2c, as reported above. In contrast, no signal was obtained with anti-MIC2 and neither with anti-SAG1 (directed against the major surface antigen of *T. gondii*), indicating that the signal obtained with anti-RONs was not due to the presence of residual membrane fragments of the parasite (data not shown). Instead, this appeared to reflect a specific association of the RONs with the host cell membrane. It is also to note that we could only rarely detect an AMA1 signal in these conditions. Indeed, quantitative analysis on three independent experiments showed that 89%±2% of the ROP1 evacuoles observed without parasites were RONs-positive while only 8.5%±2% of the ROP1 evacuoles observed without parasites were positive using the anti-AMA1 ectodomain B3.90 mAb [9] (Figure 5A), strongly suggesting that the RONs and AMA1 associate with different membranes.

Second, IFA of HFF cells pulse-invaded for 15 min showed the presence of the PVM marker ROP1 on empty vacuoles (Figure 5B). The same labelling was observed with anti-ROP2 that labelled another associated PVM rhoptry protein (data not shown). Empty vacuoles represented 6%±2% (mean±SEM of 7 independent experiments) of the total vacuole numbers when invasion was synchronized using a K^+^ buffer shift [23] and corresponded mainly to early egress of the parasite. We then checked the presence of the MJ scar on the PVM of these empty vacuoles. The association of the RONs with the PVM was systematically observed by the immunodetection of RON4/5/8 (but not RON2) on empty PVs labelled with the PVM marker ROP1 but devoid of any parasite (Figure 5B and Figure S6). Since the PV derives from the host cell membrane, this also shows that RONs are associated with the host cell membrane during invasion and probably maintained together as a complex, even after the PVM has pinched off from the host cell membrane.

### RON4, RON5, and RON8 are associated with the cytoplasmic face of the host cell membrane

We sought to address the topology of the RONs at the MJ. To this end, we first analyzed by IFA if the characteristic ring-like pattern of the RONs on invading parasites could be detected in the absence of any permeabilization (which was verified by the absence of labelling of the PVM with anti-ROP2 or anti-ROP1 sera). Since we had observed that the use of formaldehyde to fix parasite during invasion could result in partial permeabilization of the host cell membrane (data not shown), we stopped the invasion process on ice instead and performed the IFA on unfixed cells at 4°C. In these conditions, the MJ complex could not be detected unambiguously with any of the anti-RONs sera. The lack of detection of the epitopes by the antibodies could suggest either a lack of accessibility within the junction, or spatial and conformational constraints or, finally, a localization of these epitopes on the cytoplasmic side of the host cell membrane.

**Figure 6:**
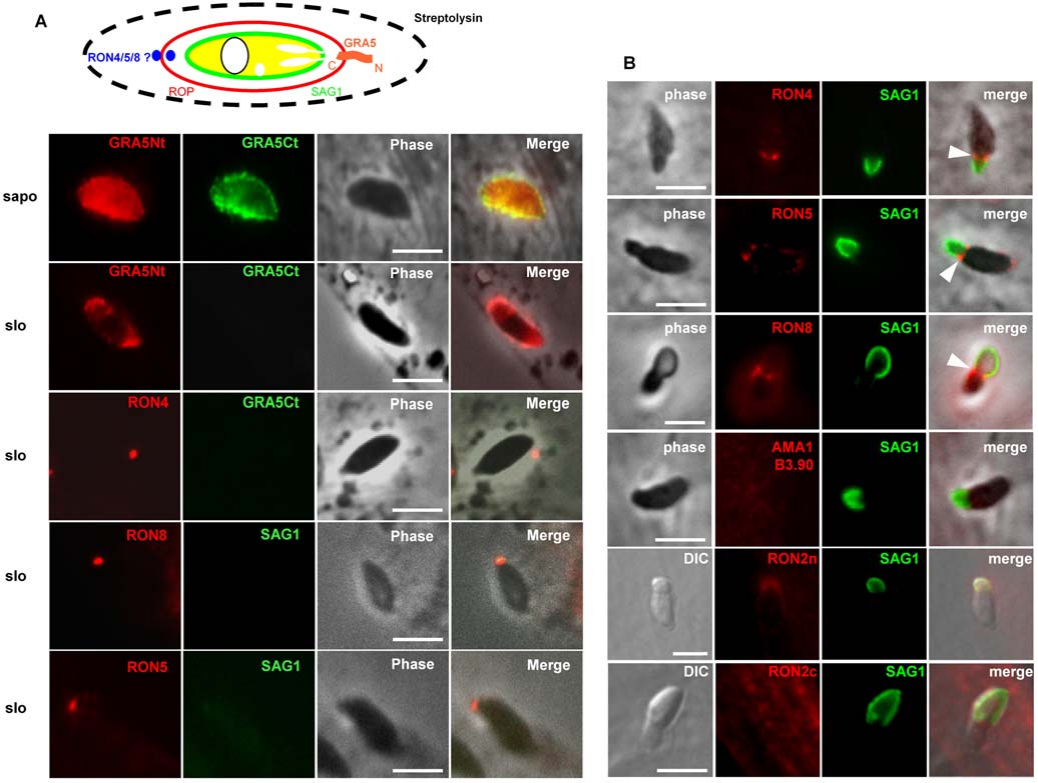
RON4, RON5, and RON8 are exposed on the cytosolic face of the host cell plasma membrane. (A) Differential solubilisation with streptolysin (schematized above) allowed for the specific labelling by IFA of epitopes exposed on the external face of PV membrane. Anti-GRA5Nt and anti-HA9 antibodies served as controls of external and internal PV sides, respectively, in a GRA5-Ct-HA9–tagged transgenic cell line. SAG1 served as a specific control for both extracellular tachyzoites and absence of permeabilization of the PVM in streptolysin-treated cells. RON4, RON5, and RON8 were detected with specific antibody on streptolysin-treated cells, indicating that they are exposed to the cytoplasmic face of the PVM. (B) Glass beads pre-loading of host cells with MJ-specific antibodies and IFA after subsequent invasion by tachyzoites reveals that RON4, RON5, and RON8 are exposed on the cytosolic face of the host cell (arrows). Cells were loaded by antibodies directed against each protein of the MJ as described in Materials and Methods and were pulse-infected for 2 min 30 s. The extracellular portion of the tachyzoites was labelled with anti-SAG1 (green), and, then, after permeabilization of the cells with saponin, the ring of the MJ was revealed by addition of the conjugate (red). DIC: differential interference contrast. Scale bar = 5 µm.

We thus addressed the possible association of RONs with the cytoplasmic face of the host plasma membrane. To this end, we examined the topology of the RONs at the MJ remnant in fully invaded parasites by differential permeabilization. Note that since RON2 was not detected at this residual junction, this approach did not allow defining the topology of RON2 in the host cell membrane. In streptolysin-O (SLO)-treated infected cells, the host cell plasma membrane was selectively permeabilized without affecting the PVM (Beckers *et al.*, 1994), allowing the selective detection of exposed cytosolic domains of PVM-associated protein (Figure 6A). These experiments were carried out with the transgenic GRA5-HA strain [24], where the HA tagged C-terminal end of the PVM marker GRA5, is exposed to the vacuolar space: hence, the absence of C-terminal labelling of GRA5 was used as control of the integrity of the PVM (Figure 6A). In addition, anti-SAG1 antibodies were used as additional control of the integrity of the PVM and to distinguish intracellular parasites (SAG1-negative) from the extracellular ones that remained attached to the cells (SAG1-positive). We first controlled that the RON scar was not detectable in the absence of streptolysin showing that it is inside the cell and not on the surface (data not shown). On samples SLO-permeabilized 15 min after invasion, RON4,5,8 (RON2 could not be detected) were found to be exposed toward the host cell cytoplasm (Figure 6A). This was also confirmed with the detection of RON4, RON5 and RON8 at the surface of isolated intact parasite-containing vacuoles (Text S1, Figure S5).

Since these two approaches allowed the detection of RONs when the parasites had fully invaded, we could not exclude that the topology of the RONs had changed during the closure of the MJ. Thus, we decided to assess the topology of these proteins during the course of invasion. To this end, we pre-loaded the host cells with anti-RONs antibodies by mechanical glass beads loading and subsequently infected the cells with *Toxoplasma* tachyzoites. In these conditions, RONs would only be detected by the pre-loaded antibodies if they were secreted into the host cell cytoplasm. The cells were then fixed during invasion, permeabilized and subjected to fluorescent secondary antibody detection. The results clearly showed the detection of the ring-shaped MJ with anti-RON4,5,8 (Figure 6B), whereas no signal was detected in the absence of permeabilization (data not shown). When cells were loaded using this technique with anti-RON2 or anti AMA1 antibodies, these proteins could not be detected at the MJ. It is to note that this approach could not be used to assess the inhibitory effect of the anti-RONs on the invasion, as the amount of antibody loaded in the cells is variable and cannot be quantified.

Taken together, our results show that the parasites can secrete the RONs complex directly into the host cell cytoplasm, RON4, RON5 and RON8 remaining associated with the cytoplasmic side of the plasma membrane/PVM during invasion, after which they persist there for a few hours as a residual structure.

## Discussion

**Figure 7:**
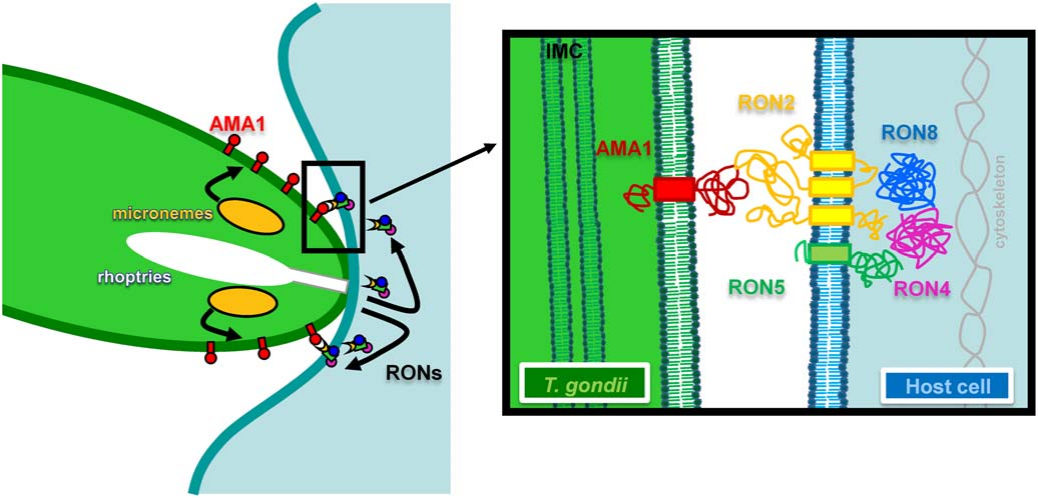
Schematic representation of the MJ organisation model. AMA1 is secreted from the micronemes at the surface of the parasite, whereas the RONs are secreted within the host cell and could serve as a receptor for AMA1 (left). Detailed view of the AMA1/RONs interaction model within the MJ is displayed on the right. A putative topology of RON2 and 5 is presented. (IMC: inner membrane complex).

The invasion process in Apicomplexa is unique among eukaryotic pathogens in that it involves a MJ structure that is used by the parasite to propel itself inside the cell using its gliding motion. Described morphologically more than 30 years ago, the composition of the structure is still poorly understood at the molecular level. A complex of four proteins AMA1/RON2/RON4/RON5 has been recently described, and two of these proteins (AMA1 and RON4) had been detected at the MJ, AMA1 being associated with the parasite membrane. We have completed the biochemical characterization of the complex and demonstrated that all the RONs are exported to the host cell membrane and, more surprisingly, three are exposed to the cytosolic face of the host cell membrane. The data we have obtained have led us to propose the first tentative model for the molecular organisation of the MJ (Figure 7).

### Insertion of material at the cytoplasmic face of the host cell membrane

The export of parasite material to the host membrane described here, particularly facing the cytoplasm of the host cell, is perfectly compatible with the thickening of the inner leaflet of the host cell membrane bilayer that has been observed at the MJ by electron microscopy [2]. It is unclear how RONs are exported into the host cell and how they could insert into, or bind to, the host cell membrane, but a secretion of rhoptry material through a transient pore in the host cell membrane has been proposed [4]. Proteins from the bulb of the rhoptry (ROPs) are known to be secreted in association with vesicles (e-vacuoles) into the host cell cytoplasm [16], but this is likely to occur after junction formation and RONs are not found in e-vacuoles. RONs must therefore be translocated at a very early stage, likely corresponding to the transient spike of conductance detected by patch clamp study of *T. gondii* invasion [25]. An association with lipids might also facilitate membrane insertion. RON4 and RON8, which are not predicted to possess a TM domain, are exposed to the cytoplasmic face of the host cell. RON5, which contains only one putative TM domain in its N-terminal end is, at least partially, exposed on this cytoplasmic face. Although we showed unambiguously that RON2 (which possesses three putative TM domains) is also a component of the MJ associated with the host cell, its precise topology at the membrane will need further studies. Indeed, whereas RON4/5/8 proteins are easily detected at the MJ, RON2 is only observed at a very early stage of junction formation or when the actin cytoskeleton is destabilized with Cyt-D, using a serum directed against a very short sequence located between the last two TM domains. This may reflect a direct or indirect interaction of this domain with the sub-plasmalemmal cytoskeleton, although invasion has been shown to critically depend on actin filaments of the parasite but not of the host cell [26]. Interestingly, only the last two TM domains of RON2 are unambiguously recognised by the prediction programs we used and the loop between these TM domains is particularly well conserved between all Apicomplexa RON2 orthologs, suggesting a conserved function.

*T. gondii* develops within a vacuole that derives from the host cell membrane. A fascinating phenomenon in Apicomplexa invasion is the selective restricted access of host proteins to the forming vacuole in which the parasite develops. This molecular sieving takes place at the MJ [2],[27]. Indeed, the presence of RONs at the cytoplasmic face of the host cell could also be involved in the exclusion of host plasma membrane proteins from the PV membrane; they would constitute a selective and protective physical barrier that would prevent protein candidates, which could mediate the fusion of the PV with the endo-lysosomal system, to be incorporated and therefore creating a non-fusigenic compartment in which the parasite could develop. These results call for functional studies to assess the respective roles of the RONs in mediating a successful invasion.

### Introducing its own receptor in the host cell membrane as a way for the parasite to circumvent host specificity?

A firm attachment of the parasite to the host cell membrane is necessary to propel it inside the PV. To do so, several scenarios might be envisaged. First, parasite ligands might be binding to host cell receptors. Another possibility is that parasite proteins, which would be directly inserted into the host cell membrane, could serve as receptors for the parasite through their extracellular domain. Our data fit perfectly with the latter scenario. Indeed, the parasite targets proteins on both membranes of the MJ; on the parasite surface for AMA1 via its TM domain [10] and on the host cell membrane counterpart, for RON2/4/5/8 (this study). In addition, we have shown that AMA1 can interact directly with RON2 *in vitro*. The domains interacting together still remain to be mapped. The precise function of AMA1 is not yet known, but several previous works showed a role of the protein in establishing close contact with the host cell suggesting that AMA1 could be involved in a receptor-ligand interaction [7],[28]. However direct binding of *Plasmodium* or *T. gondii* AMA1 to the target cell has not been proven unambiguously. Here we propose that the interaction of AMA1 with the host cell could be mediated by a RON2 receptor inserted into the host cell membrane (Figure 7). This model may account for the conserved mechanism of invasion by Apicomplexa. This type of secretion by a pathogen of a receptor for its own invasion machinery is reminiscent of the translocated intimin receptor (TIR) exported by enteropathogenic *Escherichia coli* [29], but it would be the first one to be characterised for a eukaryotic pathogen.

One crucial question is which protein is dragging the MJ backward during invasion? AMA1 does not possess the critical tryptophan that is necessary for interaction of its C-terminus with the sub-membranous motor [30]. In addition, during invasion, AMA-1 is present at the MJ but the majority of AMA1 is clearly on the parasite surface, on both sides of this adhesion zone [5],[19], implying that at least part of the AMA1 pool is not translocated posteriorly as opposed to other microneme proteins. Consistent with this notion, it is possible that part of the AMA1 pool serves as the anchor for the junction, but that another microneme TM protein interacts with the complex once assembled to propel it backwards in a glideosome-dependent motion.

Apicomplexan parasites show a wide range of host cell specificities that may depend on the expression of the MIC repertoire that differs greatly between parasites or stages of the same parasite; we hypothesize that the conserved process of invasion itself (i.e. MJ-dependent host cell entry) would be rather mediated by the specific protein complex described here, which is mostly conserved among Apicomplexa. However in this study we have characterized a new member of the MJ complex, RON8, which, in contrast to other MJ members, is specific to *T. gondii* and *N. caninum*. This highlights the fact that the MJ complex could have a different composition in several Apicomplexa and would suggest that some MJ partners could also account for driving the specificity to the host cell type.

### Distinct timing of biosynthesis between MICs and RONs

The invasion by apicomplexan parasites is a well-orchestrated mechanism involving the targeting of interacting proteins from two distinct compartments. One intriguing question is how the micronemal protein AMA1 and the RONs complex, which move through a conventional eukaryotic secretory pathway involving the rough ER, the Golgi apparatus and endosome-like structures, avoid interacting before secretion. Here we showed that even if they could physically interact as pro-proteins in cell extracts, it is probably not the case in these intermediate compartments because of a distinct timing of biosynthesis between MICs and RONs. Indeed, all the RONs (in addition to the ROPs, data not shown) are present at the same time in the pre-rhoptries in dividing parasites, whereas newly-synthesized MIC3 and M2AP (their immature forms) are not yet detected in these parasites. They are synthesized later, when the rhoptry compartment has been fully loaded.

This is the first study showing unambiguously that MICs and RONs are not expressed at the same time, which indicates that the biogenesis of rhoptries and micronemes is asynchronous in *T. gondii*, as previously suggested by ultrastructural analysis of other Apicomplexa such as *P. berghei* [31]. This distinct timing of biogenesis for proteins destined to two secretory organelles could be a general mechanism of segregation used by the parasite for interacting proteins, which would allow interaction only after secretion and during invasion.

In summary, this study extends significantly our understanding of the MJ formation and composition. The finely-tuned rhoptry and micronemal protein biosynthesis, the cooperation of these proteins originating from two different secretory organelles and the secretion of MJ components directly into the host cell, highlight the sophisticated strategies driving the active invasion of the Apicomplexa.

## Materials and Methods

The primers, antibodies and recombinant proteins generated in this study are described in Text S1.

### Host cells and parasite cultures

All *T. gondii* tachyzoites were grown in human foreskin fibroblasts (HFF) or Vero cells grown in standard condition. Tachyzoites of the RH *hxgprt-* strain of *T. gondii* deleted for hypoxanthine guanine phosphoribosyl transferase (ΔHX strain) [32] and GRA5-HA [24] were used throughout the study.

### Molecular cloning of RON8

Total RNA was isolated using RNAqueous (Ambion), according to the manufacturer's instructions. cDNA was synthetized from RH *hxgprt-* parasites using random hexamers and SuperScript II (Invitrogen) or using the SMART RACE cDNA Amplification Kit (Clontech Laboratories, Inc). cDNA fragments of TwinScan_2001 were amplified using a set of primers ML208/ML162, ML209/ML165, ML211/ML210, ML212/ML213 and ML214/ML215, and cloned into the pCR-Blunt II-TOPO vector or into the pCR-2.1-TOPO vector (Invitrogen). After sequencing, the complete open reading frame of RON8 was reconstituted from the overlapping cDNA sequences.

### Immunofluorescence

For IFA of intracellular parasites, confluent HFF monolayers were infected with RH tachyzoites for 24 h, then fixed for 30 min in 4% paraformaldehyde (PAF) in PBS. For methanol fixation, monolayers were immersed in methanol for 6 min at −20°C before IFA. After three washes in PBS, cells were permeabilized with 0.2% Triton X-100 in PBS for 10 min, blocked with 10% fetal bovine serum in PBS (PFBS) for 30 min. The cells were stained with primary antibody diluted in 10% PFBS for one hour, washed and then incubated with secondary antibody coupled to Alexa 594 or Alexa 488 (Sigma).

IFA of invading parasites were obtained by synchronisation of invasion at 4°C [6] or using a K^+^ buffer shift [23]. Invasion was carried out for 2 min30 and was stopped and fixed by adding an excess volume of 4% PAF in PBS. After three washes in PBS, cells were permeabilized with 0.05% saponin (w/v, Sigma) in PBS for 10 min, then IFA was performed as described above. Alternatively, invasion was stopped on ice, and live cells were incubated for 1 h on ice with primary antibodies, before fixation in 4% PAF, saponin permeabilization and incubation with conjugates. When needed, invasion was blocked with Cyt-D treatment by incubation of extracellular parasites with 1 µM of drug for 20 min at 37°C before invasion and then incubation of parasites for 20 min at 37°C in the presence of the drug. SLO permeabilization was conducted as described previously [33].

The coverslips were mounted onto microscope slides using Immunomount (Calbiochem). Observations were performed on a Leica DMRA2 microscope equipped for epifluorescence and images were recorded with a COOLSNAP CCD camera (Photometrics, Tucson, AZ) driven by Metaview (Universal Imaging Co., Downington, PA). Image acquisition was performed on workstations of Montpellier RIO imaging facility.

### Glass beads loading of antibodies into host cells

Loading of antibodies into the host cell was done as described previously [34]. Acid-washed 150–212 µm glass beads (Sigma) were washed 3 times with distilled water. 0.1 mg of beads were then resuspended in 300 µl of the appropriate medium containing the antibody of interest (ie. hybridoma culture supernatant, or antiserum diluted 1/30). HFF cultures growing on coverslips in a 24 wells-plate were washed twice with Hanks' Balanced Salt Solution (HBSS) before the antibodies-beads solution was put into each well. The beads were rolled onto the coverslip by tilting the plate ∼10 times, until evenly distributed over its surface. The coverslip was then transferred to another well where it was washed 3 times with HBSS and returned to DMEM culture medium and allowed to recover at 37°C and 5% CO_2_ for 30 minutes. Invasion assays were then carried out by allowing *T. gondii* tachyzoites to sediment on the HFF for 20 minutes at 4°C and subsequently warming them during 2–5 min at 37°C to trigger invasion. Invasion was stopped and cells were fixed by adding an excess volume of 4% PAF in PBS. The extracellular portion of the tachyzoites was labelled with mAb T3 1E5 specific for the surface protein SAG1. Parasites and cells were then permeabilized with saponin and incubated with anti-RONs or anti-AMA1antibodies.

### Metabolic labelling, pulse-chase analysis, immunosorption procedure

Heavily infected HFF monolayers were incubated in methionine and cysteine-free DMEM (Invitrogen) containing 4% dialyzed FCS for 30 min at 37°C in a 5% CO2 incubator prior to the addition of 50 µCi/ml [^35^S]-methionine/[^35^S]-cysteine (700 Ci/mM; Perkin Elmer) with or without BFA (5 µg/ml). The infected monolayers were then labelled for 15 or 20 min, rinsed with complete DMEM containing 10% FCS, and either processed or incubated in this medium complemented or not with BFA (5 µg/ml) for 1 h chase prior to IP. Parasite solubilization in 1% NP40 or in 0.6% SDS and immunosoption procedures were done as described previously [6],[35]. Elution was performed during 5 min at 95°C with electrophoresis sample buffer. After SDS-PAGE, the gel was impregnated with Amplify (Amersham), dried, and exposed to Biomax film (Kodak) at −80°C.

### Protein identification by mass spectrometry

Individual bands from Coomassie stained SDS-PAGE gels were excised, treated with trypsin, and extracted for analysis by nanoflow HPLC-nano-electrospray ionization on a Bruker Esquire 3000+ ion trap mass spectrometer coupled with a LC-Packings HPLC as described previously [6].

## Supporting Information

Figure S1 Specificities of anti-RONs antibodies and of RONs interactions. (A) Western Blot analysis of *T. gondii* tachyzoites lysates in non-reduced (NR) and reduced (R) conditions with specific anti-RONs antibodies and their respective pre-immune (pi) sera. Proteins of interest are shown by arrowheads. Molecular masses (kDa) are indicated on the left side and are the same for all the panels. (B) The MJ protein complex disssociates in the presence of 0.6% SDS. Western blot analysis of *T. gondii* lysates with antibodies specific for MJ proteins, before (left panel) or after (middle and right panels) immunoprecipitation with anti-RON8, RON5, and RON4n respective antibodies after 0.6% SDS lysis. Molecular masses (kDa) are indicated on the left and are the same for all the panels. * denotes non-specific bands revealed by alkaline phosphatase-conjugated secondary antibodies (conj).
(3.24 MB TIF)

Figure S2 The 30 kDa fragment is a part of RON5. (Left) Western blot analysis with anti-RON5 antibody showing that both the 110 and 30 kDa bands (arrowheads) were affinity-purified with the anti-RON5 antibody in 0.6% SDS conditions. Proteins that did not bind to the resin were collected (Flow Through: FT). (Right) Proteins isolated on the RON8 immunosorbents in 1% NP-40 conditions preserving the MJ complex were separated on SDS-PAGE, transferred to nitrocellulose, and probed with secondary antibody conjugate alone (Conj), with anti-RON8, or with anti-RON5. Both fragments of RON5 (110 and 30 kDa) were also affinity-purified with anti-RON8 in 1% NP-40. Conj: alkaline phosphatase-conjugated secondary antibody control. Molecular masses are indicated.
(0.85 MB TIF)

Figure S3 Ty-tagging of RON4 leads to the accumulation of the non-mature form of the protein in the parasitophorous vacuole. (A) Intracellular parasites expressing Ty-tagged RON4 were processed for IFA after permeabilization with triton X-100 using anti-Ty to label the tagged protein of interest and anti-ROP1 to label the rhoptries. (B) Western blot analysis of cell extracts from control HX parasites or Ty-RON4-expressing transgenic parasites. Anti-RON4 monoclonal (left) labels mature and non-mature RON4 as well as the non-mature form of the Ty-tagged version of the protein in the transgenic cell line. Anti-Ty (right) specifically labels the tagged RON4 protein.
(1.04 MB TIF)

Figure S4 Antibody against RON8 pro-peptide specifically recognizes the non-mature form of RON8. (A) Immuno-precipitation after pulse-chase (P, C) metabolic labelling of RON8 in the absence or presence of BFA (to prevent maturation of RON8) shows that the anti-proRON8 antibody immuno-precipitates the non-mature form of RON8 specifically. *T. gondii*-infected fibroblasts were labelled for 15 min with [35S] methionine/cysteine and either harvested (P, pulse) or chased for 1 h in the presence or not of BFA (C, chase). Then, the lysate was immunoprecipitated with antibody indicated, and products were run in SDS-PAGE in reduced conditions before autoradiography. The processing of RON8 was inhibited by BFA. The anti-proRON8 immunoprecipitated the immature form of RON8 in the pulse, and few immature RON8 persisted after chase as shown by the faint band of proRON8 recovered in lane C, confirming that the processing of RON8 is almost complete after one hour of chase (Figure 4A). (B) Dual immunofluorescence with anti-proRON8 and anti-RON8 antibody shows a parasite where the pro-RON8 signal overlaps partly with the ROP8 one, on two sets of punctuate structures located in the median part of an enlarged parasite undergoing endodyogeny, whereas the dots showing only the ROP signal are in a distal area corresponding to the rhoptry necks.
(1.23 MB TIF)

Figure S5 RONs are exposed on the cytoplasmic side of the PVM. Parasite-containing vacuoles were purified from infected host cells by fractionation and processed for immuno-fluorescence analysis. Negative (*T. gondii* surface protein SAG1) and positive (PVM-associated protein GRA3) controls were used to monitor the integrity of the PVM in the conditions used. Exposure of RON4, RON5, and RON8 to the outside of the vacuole was assessed using Anti-RON4 Mab, anti-RON5, and anti-RON8 antibodies. Scale bar = 5 µm.
(1.45 MB TIF)

Figure S6 RON4, RON5, and RON8 remain associated with empty parasitophorous vacuoles. IFA on HFF cells pulse-invaded for 15 min, permeabilized with saponin, and incubated with anti-RON4, anti-RON5, and anti-RON8 antibodies, detected these RONs at a residual structure associated both with parasite-containing vacuoles (arrows) and empty vacuoles (arrowheads). Scale bar = 5 µm.
(2.31 MB TIF)

Table S1 Peptides identified
(0.03 MB DOC)

Text S1 Supplementary materials
(0.07 MB DOC)
