# Supplementary figures and images for: Export of a Toxoplasma gondii Rhoptry Neck Protein Complex at the Host Cell Membrane to Form the Moving Junction during Invasion
Source: PLoS Pathog. 2009 Feb 27;5(2):e1000309. doi: 10.1371/journal.ppat.1000309 (PMC2642630; doi:10.1371/journal.ppat.1000309)

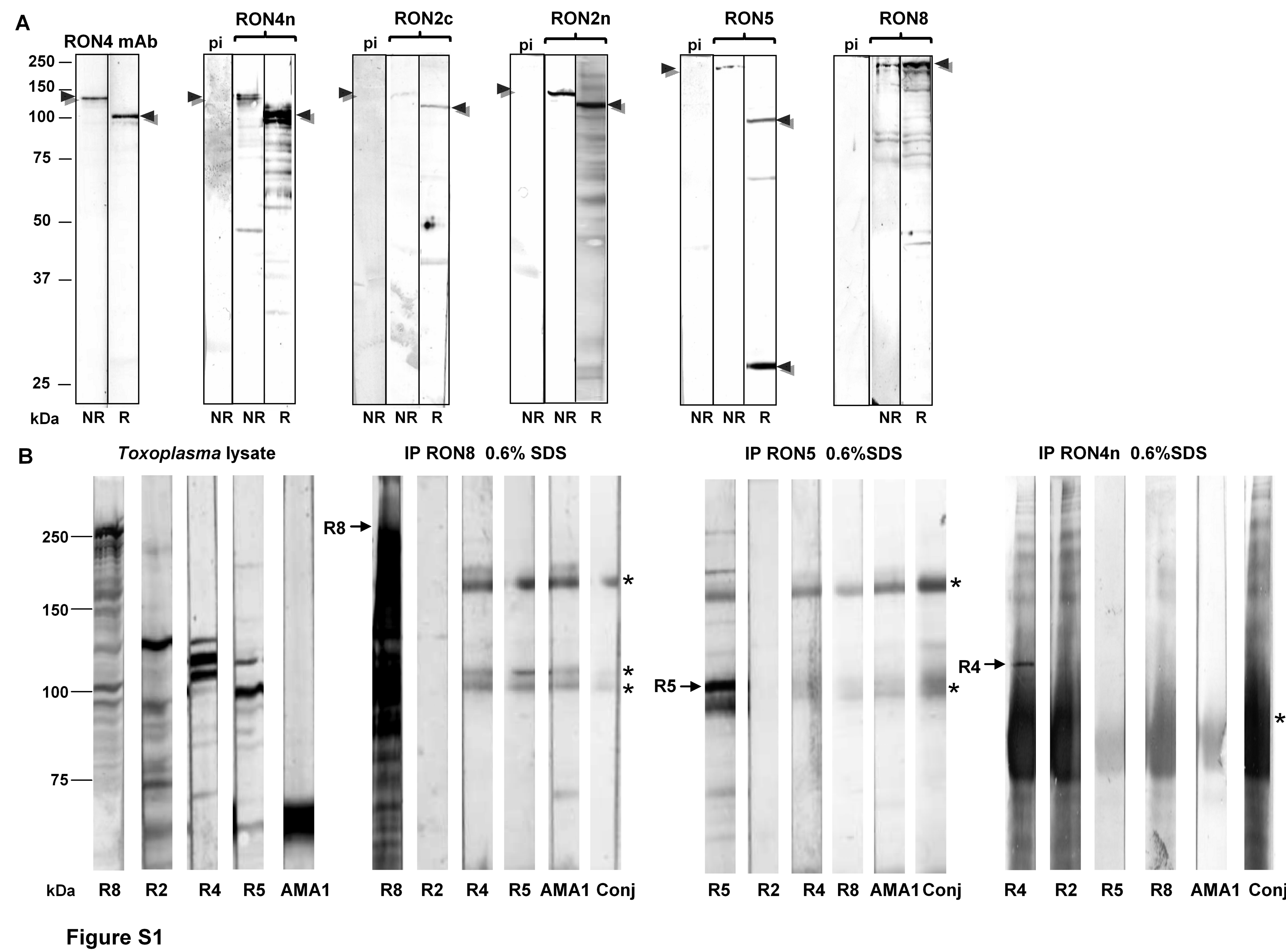

Supplement: Figure S1 — Specificities of anti-RONs antibodies and of RONs interactions. (A) Western Blot analysis of T. gondii tachyzoites lysates in non-reduced (NR) and reduced (R) conditions with specific anti-RONs antibodies and their respective pre-immune (pi) sera. Proteins of interest are shown by arrowheads. Molecular masses (kDa) are indicated on the left side and are the same for all the panels. (B) The MJ protein complex disssociates in the presence of 0.6% SDS. Western blot analysis of T. gondii lysates with antibodies specific for MJ proteins, before (left panel) or after (middle and right panels) immunoprecipitation with anti-RON8, RON5, and RON4n respective antibodies after 0.6% SDS lysis. Molecular masses (kDa) are indicated on the left and are the same for all the panels. * denotes non-specific bands revealed by alkaline phosphatase-conjugated secondary antibodies (conj). (3.24 MB TIF) [file ppat.1000309.s001.tif]

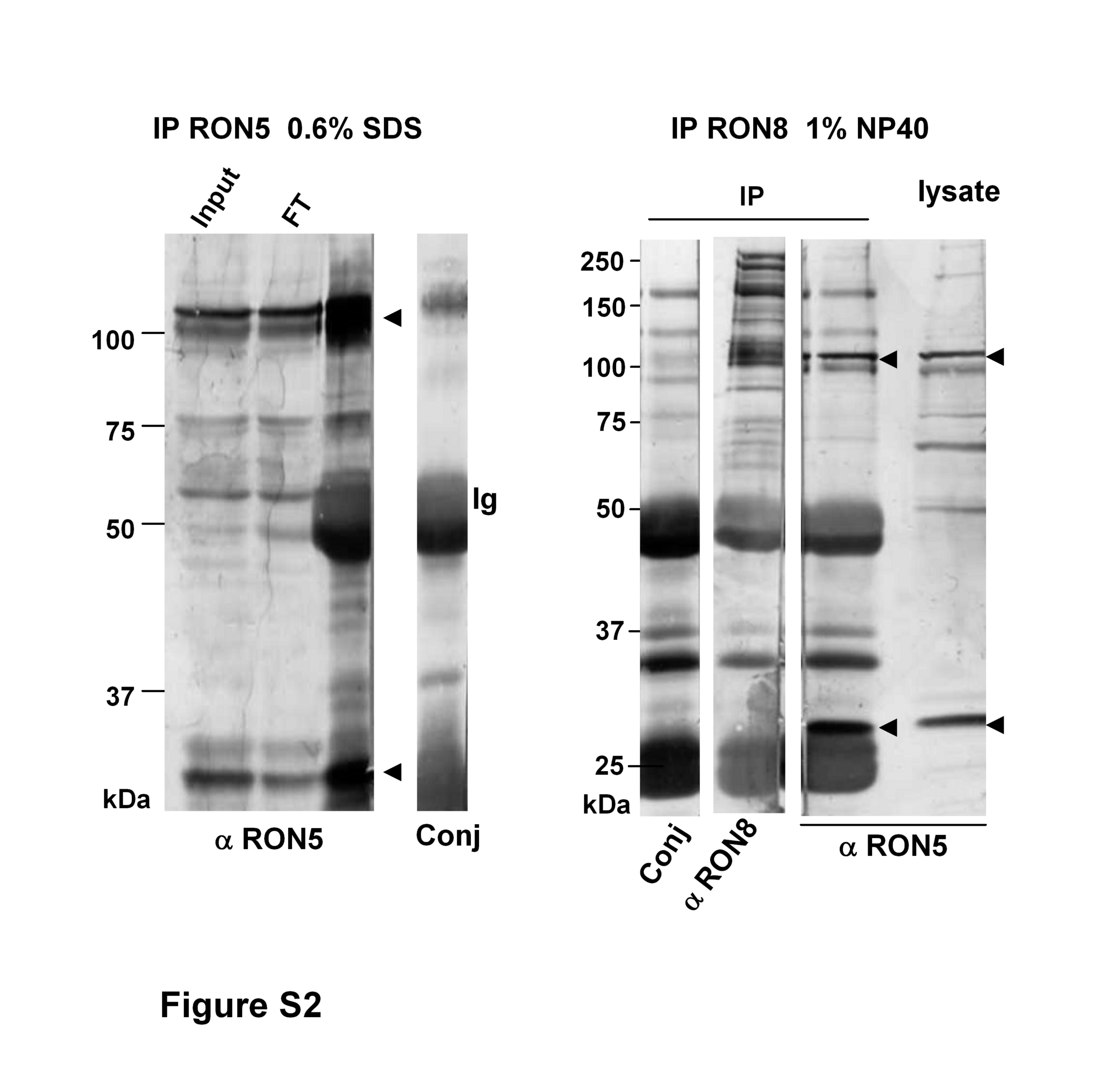

Supplement: Figure S2 — The 30 kDa fragment is a part of RON5. (Left) Western blot analysis with anti-RON5 antibody showing that both the 110 and 30 kDa bands (arrowheads) were affinity-purified with the anti-RON5 antibody in 0.6% SDS conditions. Proteins that did not bind to the resin were collected (Flow Through: FT). (Right) Proteins isolated on the RON8 immunosorbents in 1% NP-40 conditions preserving the MJ complex were separated on SDS-PAGE, transferred to nitrocellulose, and probed with secondary antibody conjugate alone (Conj), with anti-RON8, or with anti-RON5. Both fragments of RON5 (110 and 30 kDa) were also affinity-purified with anti-RON8 in 1% NP-40. Conj: alkaline phosphatase-conjugated secondary antibody control. Molecular masses are indicated. (0.85 MB TIF) [file ppat.1000309.s002.tif]

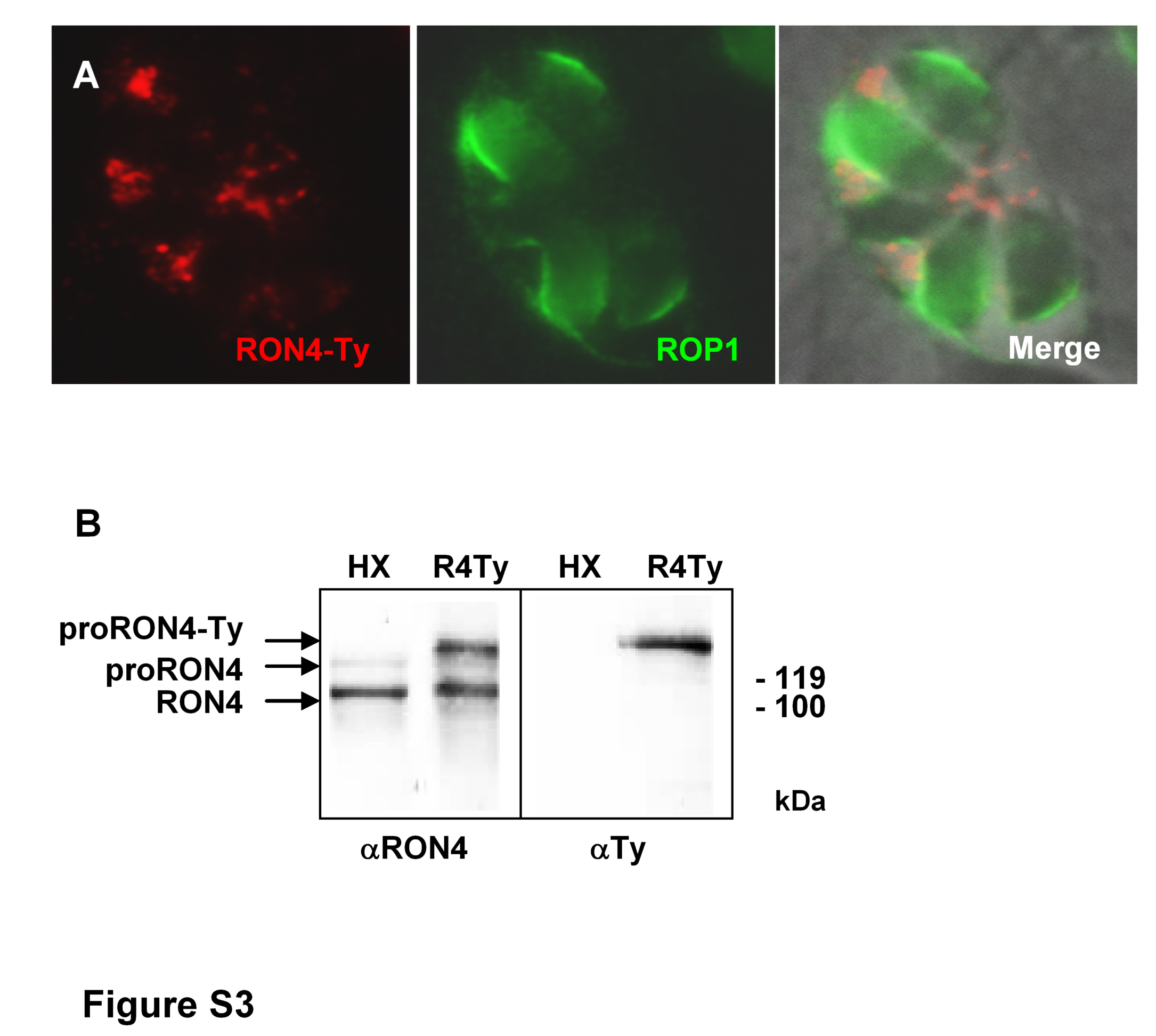

Supplement: Figure S3 — Ty-tagging of RON4 leads to the accumulation of the non-mature form of the protein in the parasitophorous vacuole. (A) Intracellular parasites expressing Ty-tagged RON4 were processed for IFA after permeabilization with triton X-100 using anti-Ty to label the tagged protein of interest and anti-ROP1 to label the rhoptries. (B) Western blot analysis of cell extracts from control HX parasites or Ty-RON4-expressing transgenic parasites. Anti-RON4 monoclonal (left) labels mature and non-mature RON4 as well as the non-mature form of the Ty-tagged version of the protein in the transgenic cell line. Anti-Ty (right) specifically labels the tagged RON4 protein. (1.04 MB TIF) [file ppat.1000309.s003.tif]

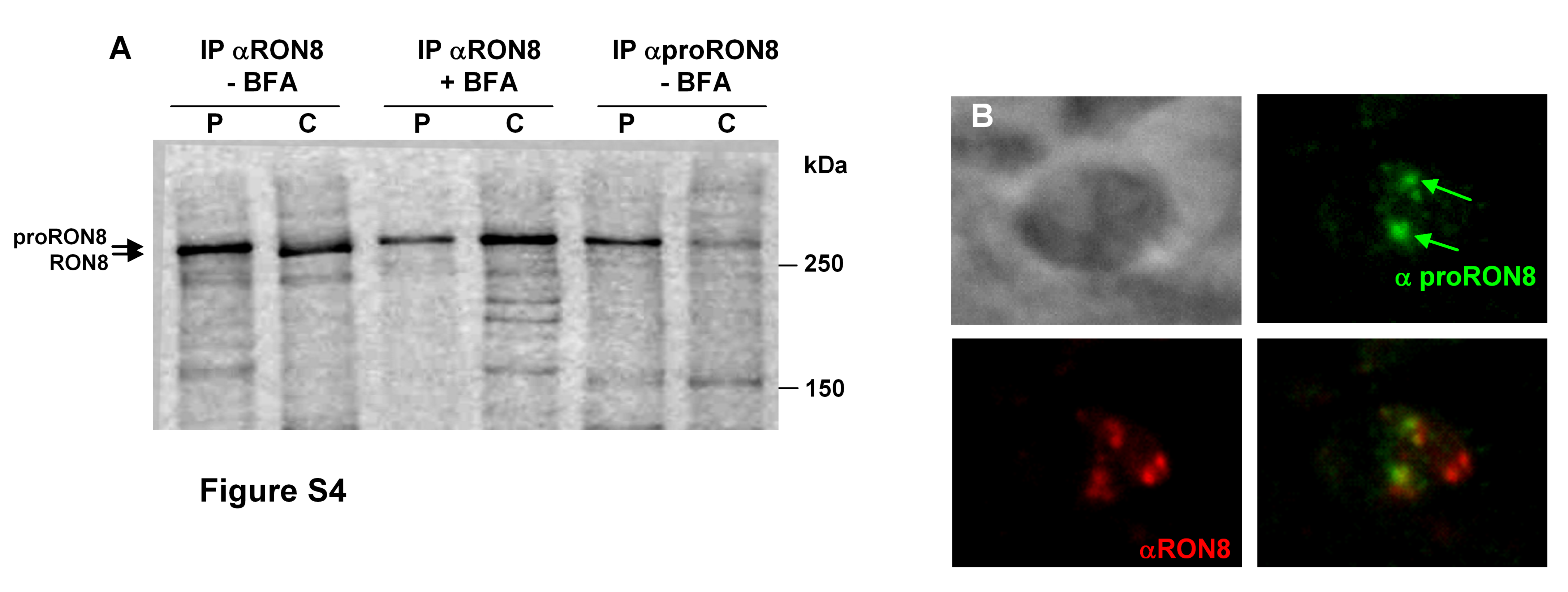

Supplement: Figure S4 — Antibody against RON8 pro-peptide specifically recognizes the non-mature form of RON8. (A) Immuno-precipitation after pulse-chase (P, C) metabolic labelling of RON8 in the absence or presence of BFA (to prevent maturation of RON8) shows that the anti-proRON8 antibody immuno-precipitates the non-mature form of RON8 specifically. T. gondii-infected fibroblasts were labelled for 15 min with [35S] methionine/cysteine and either harvested (P, pulse) or chased for 1 h in the presence or not of BFA (C, chase). Then, the lysate was immunoprecipitated with antibody indicated, and products were run in SDS-PAGE in reduced conditions before autoradiography. The processing of RON8 was inhibited by BFA. The anti-proRON8 immunoprecipitated the immature form of RON8 in the pulse, and few immature RON8 persisted after chase as shown by the faint band of proRON8 recovered in lane C, confirming that the processing of RON8 is almost complete after one hour of chase (Figure 4A). (B) Dual immunofluorescence with anti-proRON8 and anti-RON8 antibody shows a parasite where the pro-RON8 signal overlaps partly with the ROP8 one, on two sets of punctuate structures located in the median part of an enlarged parasite undergoing endodyogeny, whereas the dots showing only the ROP signal are in a distal area corresponding to the rhoptry necks. (1.23 MB TIF) [file ppat.1000309.s004.tif]

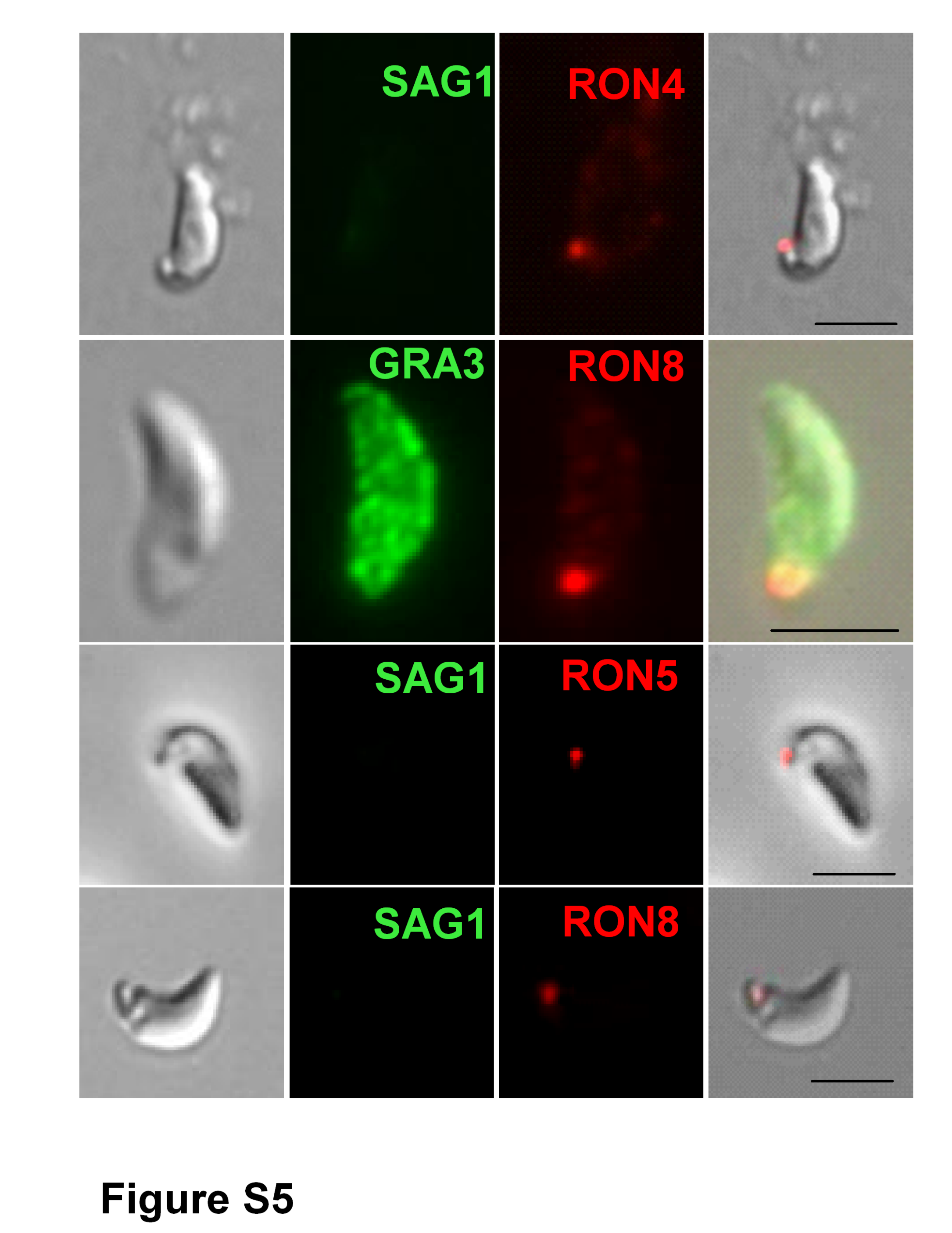

Supplement: Figure S5 — RONs are exposed on the cytoplasmic side of the PVM. Parasite-containing vacuoles were purified from infected host cells by fractionation and processed for immuno-fluorescence analysis. Negative (T. gondii surface protein SAG1) and positive (PVM-associated protein GRA3) controls were used to monitor the integrity of the PVM in the conditions used. Exposure of RON4, RON5, and RON8 to the outside of the vacuole was assessed using Anti-RON4 Mab, anti-RON5, and anti-RON8 antibodies. Scale bar = 5 µm. (1.45 MB TIF) [file ppat.1000309.s005.tif]

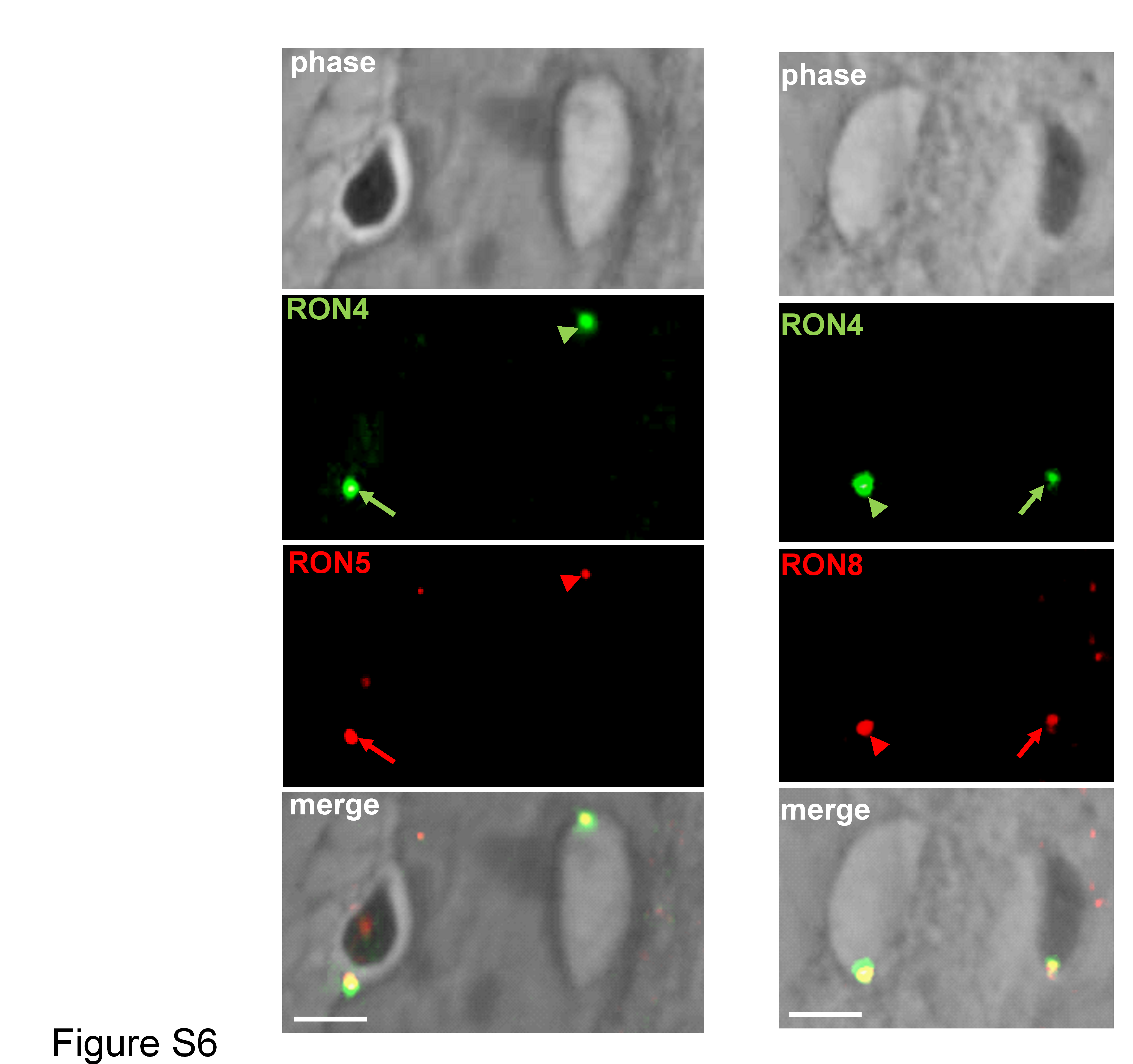

Supplement: Figure S6 — RON4, RON5, and RON8 remain associated with empty parasitophorous vacuoles. IFA on HFF cells pulse-invaded for 15 min, permeabilized with saponin, and incubated with anti-RON4, anti-RON5, and anti-RON8 antibodies, detected these RONs at a residual structure associated both with parasite-containing vacuoles (arrows) and empty vacuoles (arrowheads). Scale bar = 5 µm. (2.31 MB TIF) [file ppat.1000309.s006.tif]
