# Supplementary material for: Export of a Toxoplasma gondii Rhoptry Neck Protein Complex at the Host Cell Membrane to Form the Moving Junction during Invasion
Source: PLoS Pathog. 2009 Feb 27;5(2):e1000309. doi: 10.1371/journal.ppat.1000309 (PMC2642630; doi:10.1371/journal.ppat.1000309)
Supplement: Table S1 — Peptides identified (0.03 MB DOC) [file ppat.1000309.s007.doc]

**Table S1**

**Gel slice peptide Predicted protein __________________________________________________________________**

1 -

2 LVGATITTTEK **TgTwinScan_2001**

TLTPQELQQLK **TgTwinScan_2001**

3 DPVALLVK RON2

4 TGVEPLVDPATNSAR RON2 VAMTDAVPSGIR RON2

TLTPQELQQLK **TgTwinScan_2001**

5 LVGATITTTEK **TgTwinScan_2001**

TLTPQELQQLK **TgTwinScan_2001**

FIVGPGENDLTK **TgTwinScan_2001**

6 -

7 -

8 EVDGTLYR AMA1

YPFVYDSK AMA1

NNFLEDVPTEK AMA1 ISPFPMELLEK AMA1 NYGFYYVDTTGEGK AMA1

NSLLPVEK Hsp70

SVSEVVLVGGSTR Hsp70

TTPSYVAFTDTER Hsp70

SNQITITNDK Hsp70

INEPTAAAIAYGLDK Hsp70 precursor

NGILSVSAVDK Hsp70 precursor

LTPEEIER Hsp70 precursor

QVLEDADLQK Hsp70 precursor

NAVVTVPAYFNDAQR Hsp70 precursor

VVTPIPASK RON4

GIYPNLDELR RON4

DPVALLVK RON2

9 NNFLEDVPTEK AMA1

ISPFPMELLEK AMA1

TTPSYVAFTDTER Hsp70

SNQITITNDK Hsp70

VVTPIPASK RON4

10 LVEEGVQR MIC1

VVTPIPASK RON4

11 YPFVYDSK AMA1

HIELQQPDRPPYR AMA1 NNFLEDVPTEK AMA1

EVDGTLYR AMA1

GVQAAHHEHEFQSDR AMA1

GIYPNLDELR RON4

LVEEGVQR MIC1

12 TGVEPLVDPATNSAR RON2

VVTPIPASK RON4

VFSAVSSIK RON5

NLVSGVHSTGDSVEVR RON5

IVNSEEIQR 60S ribosomal protein

APISELPK Thioredoxin

13 VFSAVSSIK RON5

AEMVTYAMAK RON5

DATVMQQEISK RON5

MNIVFK MIC3

GYGFTTSAEK Actin

VVTPIPASK RON4

VVADPTAYGEIFER RON2

TLVEALDTMEAPK Elongation factor

14 -

15 DATVMQQEISK RON5

ALPDGLMATDVSGLDR AA_tRNA ligase

Fvqqentvqqaqtdar AA_tRNA ligase

**AATVAAGNELFK TgTwinScann_0092**

**VDEVLSMVPADPFR TgTwinScann_0092**

LTGMAFR GAPDH

VIMSAPPK GAPDH

YEDIVAAVK GAPDH

LVELAHYMSVQDGA GAPDH

DDTPMFVMGVNNDQYK GAPDH

VVDLLAPYAK ATP synthase

GIYPNLDELR RON4

ALSVPELTQQMFDAK Beta tubulin

AVLTIPHDNFPEAQK SAG1 related sequence

VAPEEHPVLLTEAPLNPK Actin

ILLNNFNFFSLNNK Ribosomal protein S3

16 VFSAVSSIK RON5

AEMVTYAMAK RON5

IGGIGTVPVGR Elongation factor

INVFYNEATGGR Beta tubulin

ALSVPELTQQMFDAK Beta tubulin

SYELPDGNIITVGNER Actin

VAPEEHPVLLTEAPLNPK Actin

VVTPIPASK RON4

GIYPNLDELR RON4

TASQSSLAPTGDNNSK RON4

YVADALSVSPR Lactate deshydrogenase

IPYGGAAPPR GRA8

LVELAHYMSVQDGA GAPDH

NLDIERPTYTNLNR Alpha tubulin

17 TASQSSLAPTGDNNSK RON4

SAEGTSESPPVPQLGTPPRPAPR RON4

AILMDLEPGTMDSVR Alpha tubulin __________________________________________________________________

Peptides recovered from mass spectrometry analysis of the RON4-associated proteins. Peptides from previously uncharacterized proteins (TgTwinScann_0092, TgTwinScann_2001) are in bold.
