# Supplementary material for: Export of a Toxoplasma gondii Rhoptry Neck Protein Complex at the Host Cell Membrane to Form the Moving Junction during Invasion
Source: PLoS Pathog. 2009 Feb 27;5(2):e1000309. doi: 10.1371/journal.ppat.1000309 (PMC2642630; doi:10.1371/journal.ppat.1000309)
Supplement: Text S1 — Supplementary materials (0.07 MB DOC) [file ppat.1000309.s008.doc]

**Supplementary Materials**

***Oligonucleotides used in this study***

ML160, 5’-CCTAGGTAAGCTCGCTAGTTTTGCTGGT-3’

ML162, 5’-GGATCCATGGCTGGGAAGTCAGAAGAGTT-3’

ML164, 5’-GGATCCATGTTGCTTGGAAATGTTCTGAA-3’

ML165, 5’-CTCGAGAAACCCTCCCCGTCGAATG-3’

ML179, 5’-CGATCGCCGGGTTTCTCGTG-3’

ML208, 5’-ATGGTGGCCACCACCCTCCA-3’

ML209, 5’-TCTTCATCTTCTTGGAAGCT-3’

ML210, 5’-ACAGGCTCGGGCAATCTGTT-3’

ML211, 5’-TCGACGGGGAGGGTTTCCGT-3’

ML212, 5’-AGTGGCAGTGTAGCCGGGAA-3’

ML213, 5’-AACAGATTGCCCGAGCCTGT-3’

ML214, 5’-TTCCCGGCTACACTGCCACT-3’

ML215, 5’-GTGCGAAGTTATTCGCTTCTG-3’

ML164, 5’-GGATCCATGTTGCTTGGAAATGTTCTGAA-3’

ML165, 5’-CTCGAGAAACCCTCCCCGTCGAATG-3’

ML224, 5’-ctgGGATCCgttgcagacatcgtgc-3’

ML225, 5’-AGTGAATTCTCAGGGCAGCATGAGGTAC-3’

ML226, 5’-gcaCATATGccggcgcctcgtccag-3’

ML227, 5’-AGTCTCGAGCTCGCGGTCGTCGAAC-3’

ML228, 5’-gcaCATATGggcaacctagtatgtg-3’

ML229, 5’-ATCGTCGACCAGATTGTGTCTCGGG-3’

ML257, 5’-CCGGAATTCGATGGTGGCCACCACCCTCCATTC-3’

ML258, 5’-CCGCTCGAGTCACTGTTGCGCGCCATCTTC-3’

ML267, 5’-CGCGGATCCAGTCCGCCAACGCCTGCT-3’

ML268, 5’-CCGCTCGAGTCAAGAAGCAGTTTTCTG-3’

ML161: 5′-AGATCTGAATTCATGGCGATAAAGAACACG-3′

ML183: 5′-ATGCATTCCTAGGAAGCTCGCTAGTT-3′

# **Antibodies**

Antibodies and dilution used in this study included an anti-Ty tag monoclonal antibody (1:200) [1], rabbit anti- HA9 (1:500 )(Covance), mAb 4A7 specific for ROP2,3,4 (1/200) [2], mAb T5 4H1 specific for RON4 (dilution 2/3 for hybridoma culture supernatants) [3], mAb T3 1E5 specific for SAG1 (p30) (1:1000) [4], mAb T7G17-113 specific of GRA5 (1:500) [5], rabbit anti-ROP1 (1:3000) and rabbit anti-ROP2 (1:200) (unpublished), rabbit anti-SAG1 (1:300) [6], mAbs specific for TgAMA1 CL22 (1:1000) [7] and B3.90 (1:300) [8], polyclonal anti N-pep AMA1 (1:40) [7], mouse anti-proMIC3 (1:1000) [9], and rabbit anti-proM2AP (1:500) [10].

***Recombinant proteins and specific antibodies***

*Rat anti-RON5*

A DNA sequence corresponding to amino acids 249 to 637 from protein RON5 (583.m00636 in the ToxoDB database: www.toxodb.org) was obtained by PCR from *T. gondii* RH tachyzoites cDNA with primers ML228 and ML229. It was then cloned into pET24a+ (Novagen) and the construct was transformed into *Escherichia coli* C41 cells to produce a recombinant protein with a C-terminal poly-histidine tag, which was used to immunize a rat.

*Rabbit anti-RON2n*

A DNA sequence corresponding to amino acids 265 to 528 from protein RON2 (Genbank [AAZ38163](http://www.ncbi.nlm.nih.gov/entrez/viewer.fcgi?db=protein&id=71559160)) was obtained by PCR from *T. gondii* RH tachyzoites cDNA with primers ML226 and ML227. It was then cloned into pET24a+ (Novagen) and the construct was transformed into *E. coli* C41 cells to produce a recombinant protein with a C-terminal poly-histidine tag, which was used to immunize a rabbit.

*Rat anti-RON2c*

A DNA sequence corresponding to amino acids 1205 to 1261 from protein RON2 (Genbank [AAZ38163](http://www.ncbi.nlm.nih.gov/entrez/viewer.fcgi?db=protein&id=71559160)) was obtained by PCR from *T. gondii* RH tachyzoites cDNA with primers ML224 and ML225. It was then cloned into pGEX-4T-3 (GE healthcare) and the construct was transformed into *E. coli* C41 cells to produce a recombinant protein with an N-terminal glutathione-S transferase tag, which was used to immunize a rat.

*Rabbit anti-RON4n*

A DNA sequence corresponding to amino acids 27 to 72 from protein RON4 (Genbank AAZ38166) was obtained by PCR from *T. gondii* RH tachyzoites cDNA with primers ML267 and ML268. It was cloned into pGEX-4T-3 (GE healthcare) and the construct was transformed into *E. coli* C41 cells to produce a recombinant protein with an N-terminal glutathione-S transferase tag, which was used to immunize a rabbit.

*Rat anti-RON8 (anti-Tw2001*)

A DNA sequence corresponding to amino acids 651 to 1281 from protein RON8 (541.m00141 in ToxoDB.org) was obtained by PCR from *T. gondii* RH tachyzoites cDNA with primers ML164 and ML165. It was then cloned into pGEX-4T-3 (GE healthcare) and the construct was transformed into *E. coli* C41 cells to produce a recombinant protein with an N-terminal glutathione-S transferase tag, which was used to immunize a rat.

*Rabbit anti-pro-RON8*

A DNA sequence corresponding to amino acids 1 to 91 from protein RON8 (541.m00141 in ToxoDB.org) was obtained by PCR from *T. gondii* RH tachyzoites cDNA with primers ML257 and ML258. It was then cloned into pGEX-4T-3 (GE healthcare) and the construct was transformed into *E. coli* C41 cells to produce a recombinant protein with an N-terminal glutathione-S transferase tag, which was used to immunize a rabbit.

His-tagged proteins were purified using a nickel-agarose column (QIAGEN) in native or denaturing condition and. GST-tagged proteins were purified by affinity purification on a gluthatione-agarose column (Sigma) according to the respective manufacturers’ instructions. When needed, a further purification was achieved by two successive SDS-PAGE and electroelutions under denaturing conditions.

Dilution of anti-RON was 1:200 for rat or rabbit anti-RON2n, 1:500 for rat anti-RON5, 1:500 for rat anti-RON8, 1:500 for rabbit anti-proRON8, 1:200 for rat anti-RON2, and 1:500 for rabbit anti-RON4n.

***Expression of a Ty-tagged RON4 protein in T. gondii***

The complete open reading frame coding for RON4 (Genbank accession number AAZ38166) was PCR-amplified using forward primer ML161 and reverse primer ML183, containing *Bgl*II and *Avr*II restriction sites, respectively, for cloning. It was then cloned into pCR-Blunt II-TOPO vector (Invitrogen) to give pZBRON4. The plasmid ptubRON4ty was designed to express the complete ORF of RON4 under the control of the tubulin promoter (TUB). It was constructed by digestion of pZBRON4 with *Bgl*II and *Avr*II to release the *RON4*-containing cassette, which was gel-purified and cloned into *Bgl*II and *Avr*II sites of ptubmic3MIC3 vector (M.L. unpublished). Transfections of parasites were conducted as described previously [11]. Overexpression of RON4 in
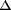
HX parasites was obtained by electroporation of 100 µg of ptubRON4ty plasmid. After overnight growth, transfectants were selected with 25 µg/ml mycophenolic acid and 50 µg/ml xanthine (for HXGPRT selection) for three passages before cloning by limiting dilution under drug selection. After expanding the clones, transfected parasites were identified by IFA with anti-ty antibodies.

***Purification of parasites-containing vacuoles***

Vero cells were infected for 15 min by *T. gondii* tachyzoites using the potassium shift synchronisation method [12], washed extensively in HBSS to remove extracellular parasites, put on ice for 5 minutes, washed twice in cold PBS and then scrapped. Parasite-containing vacuoles were freed by passing infected Vero cells through a 22 gauge needle 4 times. The resulting material was pelleted at 800 g for 10 minutes at 4°C and processed for immunofluorescence. The primary antibody was put in contact with vacuoles containing live cells in DMEM with 5% FCS for 45 minutes at 4°C. Vacuoles were washed 3 times in PBS, fixed for 20 minutes with 4% PAF at room temperature and incubated with secondary antibody at room temperature. Vacuoles were left to adhere on poly-lysine-coated slides before observation with the microscope.

Supplemental references

1. Bastin P, Bagherzadeh Z, Matthews KR, Gull K (1996) A novel epitope tag system to study protein targeting and organelle biogenesis in Trypanosoma brucei. Mol Biochem Parasitol 77: 235-239.

2. Sadak A, Taghy Z, Fortier B, Dubremetz JF (1988) Characterization of a family of rhoptry proteins of *Toxoplasma gondii*. Mol Biochem Parasitol 29: 203-211.

3. Leriche MA, Dubremetz JF (1991) Characterization of the protein contents of rhoptries and dense granules of *Toxoplasma gondii* tachyzoites by subcellular fractionation and monoclonal antibodies. Mol Biochem Parasitol 45: 249-259.

4. Couvreur G, Sadak A, Fortier B, Dubremetz JF (1988) Surface antigens of *Toxoplasma gondii*. Parasitology 97 ( Pt 1): 1-10.

5. Charif H, Darcy F, Torpier G, Cesbron-Delauw MF, Capron A (1990) *Toxoplasma gondii*: characterization and localization of antigens secreted from tachyzoites. Exp Parasitol 71: 114-124.

6. Harning D, Spenter J, Metsis A, Vuust J, Petersen E (1996) Recombinant *Toxoplasma gondii* surface antigen 1 (P30) expressed in Escherichia coli is recognized by human *Toxoplasma*-specific immunoglobulin M (IgM) and IgG antibodies. Clin Diagn Lab Immunol 3: 355-357.

7. Hehl AB, Lekutis C, Grigg ME, Bradley PJ, Dubremetz JF, et al. (2000) *Toxoplasma gondii* homologue of plasmodium apical membrane antigen 1 is involved in invasion of host cells. Infect Immun 68: 7078-7086.

8. Donahue CG, Carruthers VB, Gilk SD, Ward GE (2000) The *Toxoplasma* homolog of Plasmodium apical membrane antigen-1 (AMA-1) is a microneme protein secreted in response to elevated intracellular calcium levels. Mol Biochem Parasitol 111: 15-30.

9. El Hajj H, Papoin J, Cerede O, Garcia-Reguet N, Soete M, et al. (2008) Molecular signals in the trafficking of *Toxoplasma gondii* protein MIC3 to the micronemes. Eukaryot Cell 7: 1019-1028.

10. Harper JM, Huynh MH, Coppens I, Parussini F, Moreno S, et al. (2006) A cleavable propeptide influences *Toxoplasma* infection by facilitating the trafficking and secretion of the TgMIC2-M2AP invasion complex. Mol Biol Cell 17: 4551-4563.

11. Reiss M, Viebig N, Brecht S, Fourmaux MN, Soete M, et al. (2001) Identification and characterization of an escorter for two secretory adhesins in *Toxoplasma gondii*. J Cell Biol 152: 563-578.

12. Kafsack BF, Beckers C, Carruthers VB (2004) Synchronous invasion of host cells by *Toxoplasma gondii*. Mol Biochem Parasitol 136: 309-311.
